# Supraspinal Mechanisms Underlying Ocular Pain

**DOI:** 10.3389/fmed.2021.768649

**Published:** 2022-02-08

**Authors:** Nicholas J. Pondelis, Eric A. Moulton

**Affiliations:** ^1^Brain and Eye Pain Imaging Lab, Pain and Affective Neuroscience Center, Department of Anesthesiology, Critical Care and Pain Medicine, Boston Children's Hospital, Harvard Medical School, Boston, MA, United States; ^2^Department of Ophthalmology, Boston Children's Hospital, Harvard Medical School, Boston, MA, United States

**Keywords:** pain, eye, neuroimaging, fMRI, supraspinal, brain, brainstem, ocular

## Abstract

Supraspinal mechanisms of pain are increasingly understood to underlie neuropathic ocular conditions previously thought to be exclusively peripheral in nature. Isolating individual causes of centralized chronic conditions and differentiating them is critical to understanding the mechanisms underlying neuropathic eye pain and ultimately its treatment. Though few functional imaging studies have focused on the eye as an end-organ for the transduction of noxious stimuli, the brain networks related to pain processing have been extensively studied with functional neuroimaging over the past 20 years. This article will review the supraspinal mechanisms that underlie pain as they relate to the eye.

## Introduction

Ophthalmology as a clinical field has a preoccupation with what can be seen, particularly for patients presenting with eye pain. The patient reports eye pain as a symptom, and the clinician collects the available data to reach a diagnosis. Data come in the form of patient reports, medical history, professional acumen, and clinical findings, such as those obtained with a slit lamp exam of the anterior and posterior segment or specialized equipment that provide intensely magnified views of ocular structures. Despite the ever-expanding options for precise clinical evaluation, pain is no guarantee of a physically observable sign of tissue damage. Pain is subjective by its very nature, and similar inputs can result in bewildering and wildly inconsistent pain responses. However, modern functional neuroimaging tools have allowed scientists to investigate this symptom in the context of the inner workings of the brain.

Pain serves as a crucial system to avoid bodily injury and damage. Pain accomplishes this function by creating strong, memorable disincentives for potentially damaging activity as well as protective reflexes and convalescence-promoting behaviors to prevent or limit damage. As defined by the International Association for the Study of Pain (IASP), it is “an unpleasant sensory and emotional experience associated with, or resembling that associated with, actual or potential tissue damage” (1). Pain is an attention-demanding, conscious state that takes precedence over other processes (2, 3). Ocular pain can be debilitating and serves to protect a critically important sensory apparatus.

Much as visual processing involves numerous brain regions, pain perception is generated by an amalgam of signals and modifications carried by a wide network of brain regions and pathways. These regions work in delicate balance with each other and are influenced by individual neurobiological variation, resulting in an inherently subjective experience (3). The transduction of noxious information travels along multiple pathways to the brain and is processed during its transmission from the periphery as well as at the highest cortical levels. The diverse inputs from the numerous supraspinal processing areas are eventually integrated. The result is the multidimensional perception of pain, with its intensity, unpleasantness, emotional connotation, and more.

### Conceptualization of Pain

Pain has been categorized into three distinct, concurrent dimensions: affective-motivational, sensory-discriminative, and cognitive-evaluative (4). The sensory and discriminative dimensions are related to the location, characteristics, intensity, and timing of a stimulus that evokes pain. The affective and motivational aspects of pain are highly intertwined with emotion and constitute the unpleasant aspects of pain that give rise to behavioral responses. The cognitive and evaluative dimension is the means by which the brain is able to comprehend and contextualize the consequences of injury or pain, anticipate pain based on memory, and inhibit or facilitate painful sensation (5, 6). Multiple distinct brain regions and networks underlie these discrete aspects of pain and their flavoring of the pain experience (7–10).

### Nociceptive vs. Neuropathic Pain

Nociception is the physiological encoding and detection of noxious stimuli by the central and peripheral nervous systems (11). Though they are often concurrent, nociception and pain can occur independently, and the terms are not directly interchangeable. Nociceptive transduction can take place during the sensation of itch, which itself is not painful; likewise, pain can exist untethered from peripheral noxious input, as with phantom limb pain (12).

Nociceptive pain “arises from actual or threatened damage to non-neural tissue and is due to the activation of nociceptors” (13), a detection and warning system for the presence of intense stimuli. The transfer of nociceptive signals through supraspinal centers to the cortex generates pain, that is, triggers avoidance reflexes, unpleasant sensations, and a negative emotional state. This multifaceted experience overrides most ongoing processes and diverts attention to the detection of, and withdrawal from, a noxious stimulus (2).

Pain may persist over long periods of time and can serve a beneficial purpose by reporting the extent of injury and progression of tissue repair while promoting convalescent behavior (14). To facilitate this, after injury the central nervous system can establish long-lasting sensitivity to peripheral inputs, which may help to prevent further harm during recuperation (15, 16). These changes do not always resolve after injury and sometimes cannot be clearly linked to disease as the source. Pain recurring or persisting for longer than 3 months is defined as chronic and may be the consequence of underlying disease (chronic secondary pain) or exist without a clear cause or insult (chronic primary pain) (17, 18).

Neuropathic pain is “a result of a lesion or disease of the somatosensory nervous system” and may be peripheral or central in nature (13). As part of the repair process after peripheral nerve injury, both damaged and healthy primary nerve fibers (but not their peripheral receptors) may fire action potentials spontaneously; the resulting ectopic pain is a natural consequence of healing but is nevertheless considered peripheral neuropathic pain (16, 19). In the case of centralized neuropathic pain, the complex balance of supraspinal mechanisms underlying the CNS's signaling and modulatory capacity can become disrupted and manifest pain without significant peripheral instigation (14, 16).

### Organizational Summary

The primary aim of this review will be to describe the functionality and role of brain structures related to pain processing in the context of human neuroimaging (20). We will first briefly summarize how the peripheral nervous system encodes and transmits ocular nociceptive signals to the central nervous system by major ascending pathways. We then will focus on neuroimaging of supraspinal structures related to pain. Finally, we will explore sensitization in these circuits and briefly discuss their manifestation at the network level.

## Sensory Innervation of the Anterior Segment

The eye contains a host of sensitive tissue, with the cornea being the most densely innervated in the body (21). Nociceptors within these areas are largely offshoots of the ophthalmic division of the trigeminal nerve, but other pathways and sensory modalities contribute to nociception and processing of the full spectrum of sensory inputs to the anterior segment and eye. These peripheral pathways have been more thoroughly described previously (22, 23). The healthy cornea is exclusively innervated by large-diameter, myelinated A-delta nerve fibers and small-diameter, unmyelinated C-fibers (22, 24). The three primary classes of corneal sensory afferents are polymodal nociceptors, mechanonociceptors, and cold thermoreceptors, each of which preferentially responds to various sensory stimuli. The polymodal C-fibers are the most abundant and can detect a wide range of stimuli, including mechanical, thermal, and chemical. Cold receptors respond to thermal changes and consist of either A-delta or C-fibers, while specific mechanoreceptors are exclusively A-delta fibers and activate upon mechanical stimulation alone. These same classes of peripheral sensory afferents have also been identified in the episclera, bulbar conjunctiva, iris, and ciliary body, while non-corneal ocular tissue, especially the eyelids, may have numerous additional types of low-threshold mechanoreceptors (21, 22, 24, 25). The trigeminal system is also responsible for the innervation of both meningeal and dural vessels, and information from peripheral receptors in these areas travels alongside other sensory information through trigeminal pathways (26, 27).

Several peripheral sensors in the anterior of the eye contain melanopsin, a photopigment that offers a light transduction mechanism that may lead to pain perception. With a peak wavelength sensitivity of 480 nm, melanopsin-based photoreception can occur in intrinsically photosensitive retinal ganglion cells (ipRGCs) and is increasingly implicated as a source for light-induced pain (28–32). These ipRGCs can generate their own signal independent of rod and cone involvement in response to light absorption yet can additionally receive or relay input from classical RGCs and support cells (33–36). In addition to ipRGCs, melanopsin has been found in a variety of other tissue in mammals and humans, including expression and prospective inherent photosensitivity in the cornea, iris, ciliary body projections, certain vasculature, and trigeminal neurons themselves (36–40). These peripheral melanopsin-containing populations can generate a light-response without traversing the optic nerve (26, 27, 40, 41).

### Nociceptive Pathways

Peripheral receptors in the anterior segment are conventionally the gateway for nociceptive transduction that leads to the experience of pain. The transfer of peripheral signals to the brain is facilitated by a number of pathways, including the trigeminothalamic pathway, the parabrachial nucleus pathway, and the melanopsin pathway.

In health, and in conjunction with their respective peripheral afferents, these pathways supply the brain with vital information regarding the health of the eye and serve as a broad and fine-tuned detection mechanism for the prevention of ocular damage. However, damage to this network can result in dysfunction of peripheral neurons, intermediaries between them and the brain, or cortical areas themselves—all resulting in pain (22). Likewise, maladaptive sensitization of these same critical nociceptive pathways can lead to unduly painful outcomes for patients. Even after direct insults to the peripheral fibers of these nerves are healed, pain can persist. Often this persistent pain involves a central component of the nociceptive pathway that can be difficult to detect, let alone resolve.

#### Trigeminothalamic Pathway

Ocular nociceptive and sensory information travel from peripheral sites through primary fibers of the ophthalmic trigeminal nerve to the ipsilateral trigeminal ganglion, where the neuronal bodies are somatotopically organized along with the other trigeminal branches. First-order neurons synapse to second-order neurons in the pons at the trigeminal brainstem nuclear complex (TBNC). The synapses of nociceptive and thermosensory neurons are located in the spinal trigeminal nucleus caudalis (spVc) transition zones. From the medullary dorsal horn, nociceptive information travels along groups of neurons to either the contralateral thalamus or the ipsilateral parabrachial nuclei (22).

**Figure 1:**
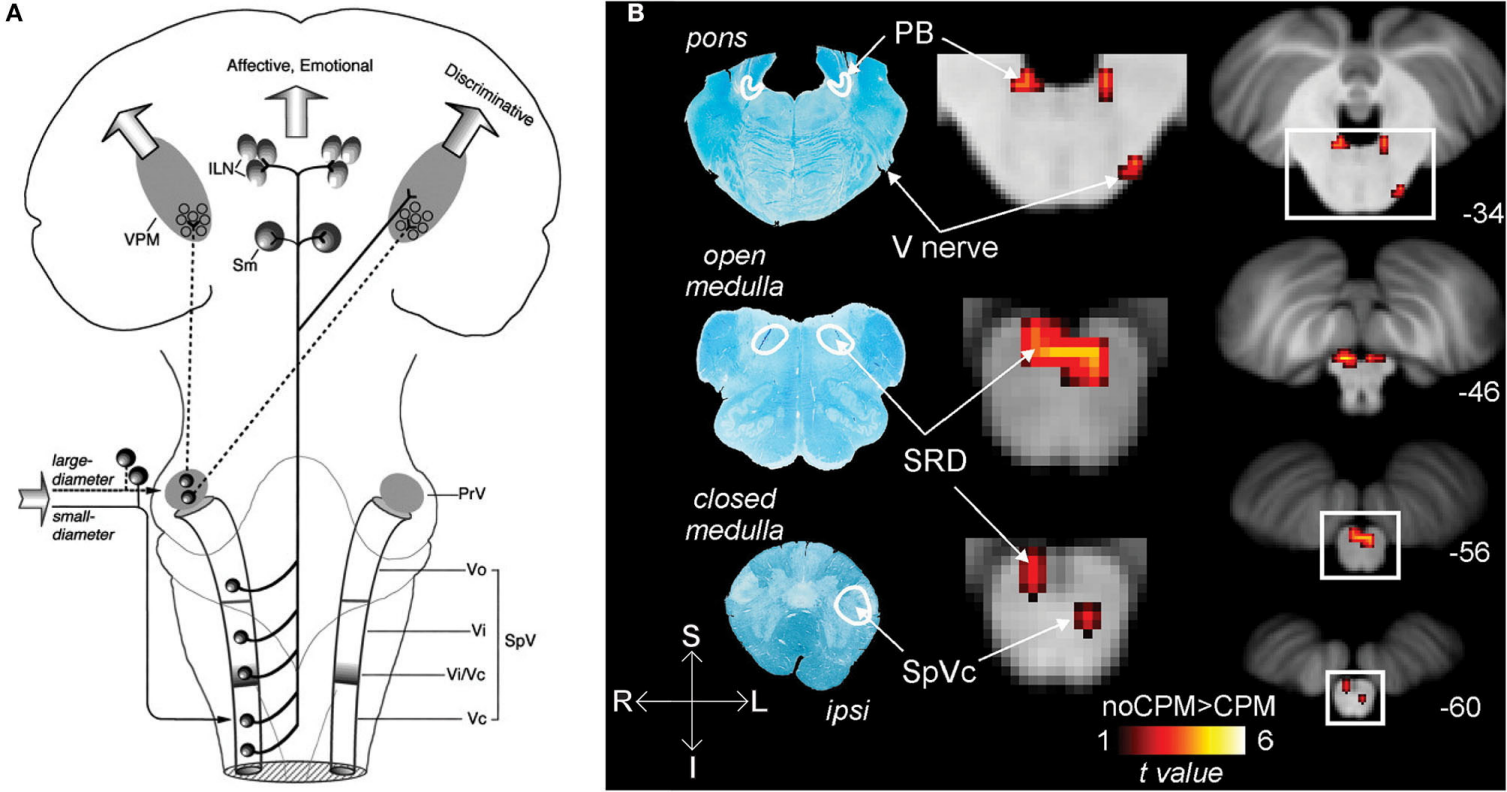
Nociceptive pathways. **(A)** The path of afferent signal transmission from the periphery to the cortex through major projections of the trigeminothalamic pathway. Reprinted/adapted by permission from Springer Nature Customer Service Centre GmbH: Springer Nature Trigeminothalamic Tract Projections. In: Schmidt R., Willis W. (eds) Encyclopedia of Pain by Ke Ren, Copyright (2007). DOI: https://doi.org/10.1007/978-3-642-28753-4_4626. **(B)** Decreased brainstem fMRI activity, including PB, during endogenous analgesia. Red/yellow indicates regions where fMRI responses to noxious stimuli demonstrated a signal decrease following conditioned pain modulation. Decreased activation was noted in the SRD, SpVc, and the trigeminal nerve along with the PB. On the left, myelin-stained sections are displayed alongside corresponding MRI images of the brainstem. Reprinted from NeuroImage, Vol 124(Part A), AM Youssef, VG Macefield, LA Henderson, Pain inhibits pain; human brainstem mechanisms, p54–62, Copyright (2020), with permission from Elsevier. DOI: https://doi.org/10.1016/j.neuroimage.2015.08.060. PrV, principal sensory nucleus; SpV, spinal trigeminal nucleus; Vo, subnuclei oralis; Vi, subnucleus interpolaris; Vc, subnucleus caudalis; PB, parabrachial nucleus; SRD, subnucleus reticularis dorsalis; SpVc, spinal trigeminal nucleus caudalis; V, trigeminal nerve; Compas: S, superior; I, inferior; R, right; L, left.

The trigeminal connections to the thalamus are involved in the sensory-discriminative and affective-motivational components of pain (4). From the TBNC, second-order neurons destined for the thalamus leave the subnucleus caudalis, decussate, and subsequently enter the contralateral anterior trigeminothalamic tract (lemniscus). The neurons then ascend to synapse with tertiary neurons in the medial and somatosensory (lateral) thalamic nuclei (42). Nociceptive information from the thalamus is then relayed to higher brain regions where it is further processed, eventually resulting in pain perception (22, 43, 44) (Figure 1A).

#### Parabrachial Nucleus Pathway

The nociceptive inputs that pass through the parabrachial nuclei (PBN) are involved in the affective-motivational and autonomic components of pain. The parabrachial nuclei are a bilateral grouping of neurons located in the brainstem at the junction of the dorsolateral pons and midbrain, surrounding the superior cerebellar peduncle (45). The PBN receive afferent input from second-order trigeminal neurons in the spVc (46). The PBN pass the information to the central nucleus of the amygdala, the hypothalamus, periaqueductal gray and RVM, and onto parts of the spino-parabrachial pathway, which innervates the anterior cingulate (ACC) and insular (IC) cortices via the thalamus (47). These findings in non-human primates have been reproduced in humans, where noxious stimuli to the orofacial region produce increased BOLD fMRI signal intensity in the spV and subsequently several other supraspinal regions, including the PBN (48).

In addition to acting as a conduit for peripheral nociceptive information, the PBN have a wide array of functions, such as autonomic modulation (49), and are involved in pain processing, mostly as a key supraspinal region for encoding the affective component of pain (50). The PBN also play a role in pain modulation, as the region is implicated in some forms of endogenous analgesia (48), and low-frequency deep brain stimulation of the PBN provides meaningful pain relief, although these findings are intertwined with stimulation of other, more canonical analgesia-associated brain regions in certain cases (51) (Figure 1B).

#### Melanopsin Pathway

Light information from ipRGCs is largely transmitted through the optic nerve until reaching target destinations in the brain. The three primary tracts that project to the brain are the retino-thalamo-cortical pathway, the retino-midbrain pathway, and the retino-hypothalamic tract. The retino-thalamo-cortical pathway is a direct connection between ipRGC populations and the pulvinar nuclei within the posterior thalamus (26, 52, 53). The retino-midbrain-parasympathetic (or retinomesencephelatic) pathway brings photic signals from the retina directly to the olivary pretectal nucleus in the midbrain (54). The retinohypothalamic tract extends through the optic nerve before synapsing to several areas, with the major target being the suprachiasmatic nucleus (36). This tract can be subdivided into three broad types of innervation: afferents leading to hypothalamic neurons directly, referred to simply as the retinohypothalamic tract; the retino-hypothalamo-parasympathetic tract, which innervates the superior salivatory nucleus in the brainstem; and the retino-hypothalamo-sympathetic tract, which connects to the intermediolateral nucleus in the spine (54).

The melanopsin involvement in light detection in ipRGCs, anterior segment structures, and nociceptive neurons has led to the exploration of pain enhancement by light along these and the trigeminal nociceptive pathways (26, 27, 40, 55–58).

## Central Representation of Pain

**Figure 2:**
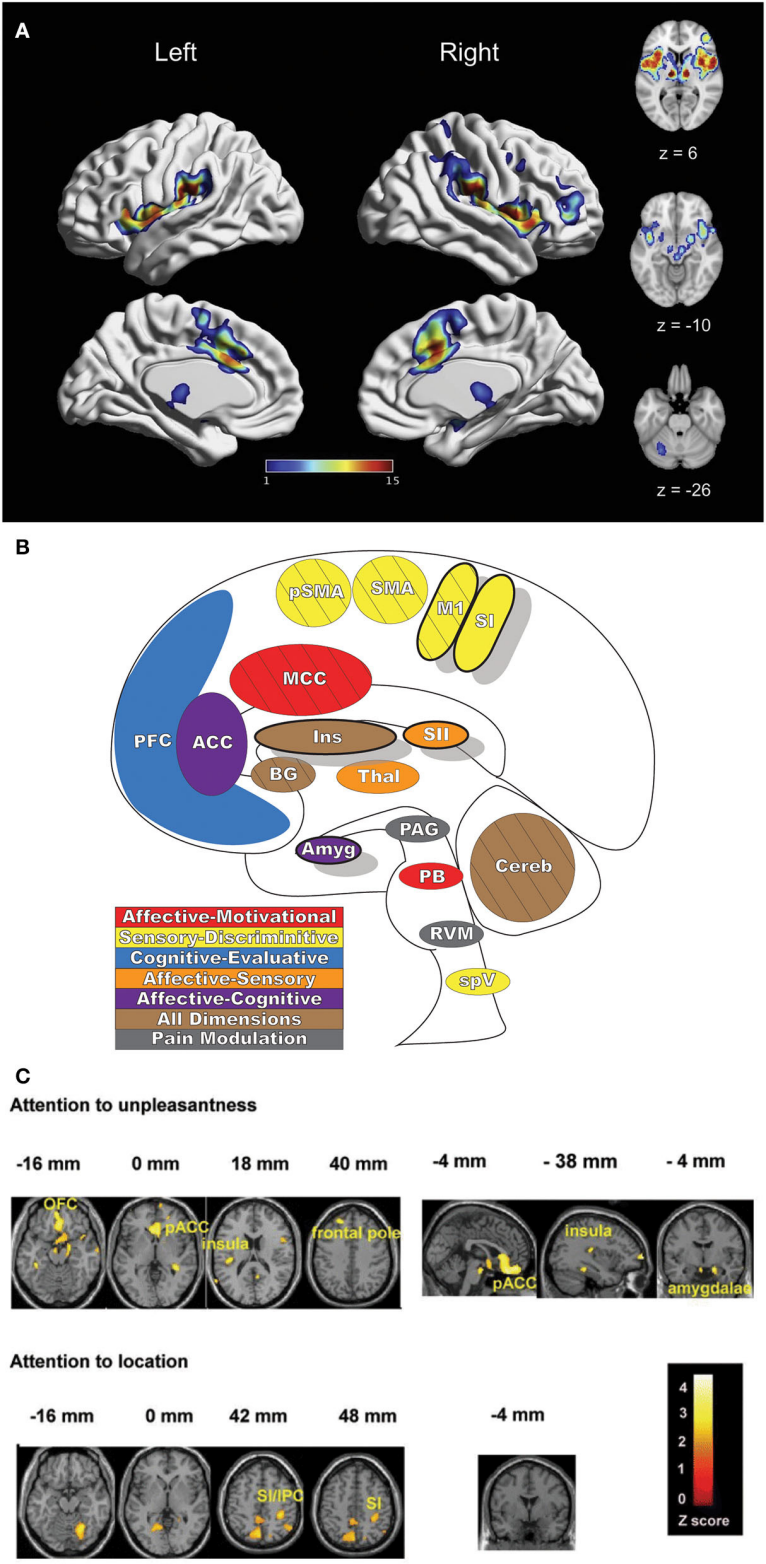
Pain-related areas in the brain and brainstem. **(A)** A meta-analysis of pain neuroimaging studies defines a set of brain regions consistently active across 222 experiments from 200 reports, including bilateral activity in the secondary somatosensory cortex, insular cortex, midcingulate cortex, and thalamus. Voxel values increase from 1 to 15 with increasing convergence across 15 total main effects meta-analyses that each reflect pain-related activation. Reprinted from Neuroscience & Biobehavioral Reviews, Vol 112, A Xu, B Larsen, EB Baller, JC Scott, V Sharma, A Adebimpe, AI Basbaum, RH Dworkin, RR Edwards, CJ Woolf, SB Eickhoff, CR Eickhoff, TD Satterthwaite, Convergent neural representations of experimentally-induced acute pain in healthy volunteers: A large-scale fMRI meta-analysis, p300–23, Copyright (2020), with permission from Elsevier. https://doi.org/10.1016/j.neubiorev.2020.01.004. **(B)** Schematic of brain areas related to the processing of the multidimensional experience of pain. Each region is color coded to correspond to its hypothesized dimension of pain, while hatch-marks indicate processing associated with pain-related movement. Thick black borders indicate regions located more lateral to the midline. Relative size of each region is roughly proportional for structures larger than SII. **(C)** Attention to different features of a painful stimulus can shift activation patterns. Focusing on the unpleasantness of pain vs its location results in different patterns of brain activation when examined by PET, providing evidence that the unique dimensions of pain may be processed in separate brain areas. Reprinted from the European Journal of Neuroscience, Vol 21(11), B Kulkarni, DE Bentley, R Elliott, P Youell, A Watson, SW Derbyshire, RS Frackowiak, KJ Friston, AK Jones, Attention to pain localization and unpleasantness discriminates the functions of the medial and lateral pain systems, p3133-42, Copyright (2005), with permission from John Wiley and Sons. DOI: https://doi.org/10.1111/j.1460-9568.2005.04098.x. SI, primary somatosensory cortex; SII, secondary somatosensory cortex; MCC, midcingulate cortex; ACC, anterior cingulate cortex; Ins, Insular Cortex; Amyg, amygdala; PFC, prefrontal cortex; M1, primary motor cortex; SMA, supplementary motor area; pSMA, pre-supplementary motor area; BG, basal ganglia; Cereb, cerebellum; PAG, periaqueductal gray; PB, parabrachial nuclei; RVM, rostral ventromedial medulla; spV, spinal trigeminal nucleus; Thal, thalamus; OFC, orbitofrontal cortex; pACC, perigenual cingulate corex; IPC, inferior parietal cortex.

Pain is a complex and multifaceted experience and, as such, a large number of cortical and subcortical supraspinal areas are involved in the interpretation of noxious stimuli and the resultant sensation of pain. The supraspinal areas most likely to be activated in response to a wide variety of noxious stimuli are the thalamus, secondary somatosensory cortex, anterior/mid-cingulate cortex, and the insula (7, 8, 10). In addition to these areas, studies with differing parameters and means of noxious stimulation have found additional brain regions that are involved in pain under certain conditions, including the primary somatosensory cortex, amygdala, lateral prefrontal cortex, primary and supplementary motor areas, pre-supplementary motor area, basal ganglia, cerebellum, and brainstem (10) (Figures 2A,B).

Distinct aspects of pain are transmitted through separate nuclei in the thalamus to higher brain structures and have been grouped into a classification scheme of medial and lateral pain systems based on the organization of innervation to and from the nuclear groupings (42). The lateral pain system is associated with the sensory-discriminative components of pain, and routes information from somatotopically arranged lateral thalamic nuclei (ventral posterior and posterior, including VPM) to the somatosensory cortices and posterior insula (42, 59–61). The medial pain system, underlying the affective-motivational pain dimension, processes and transfers pain information from non-somatotopically-organized medial dorsal, midline, and intralaminar thalamic nuclei to the cingulate cortex, prefrontal cortex, amygdala, and hypothalamus, and their subsequent projections to descending modulatory areas (42, 60, 62). As pain is an incredibly salient experience, many of these brain regions are involved not just in nociception, but also in attention and motor-response networks (9, 63) (Figure 2C).

### Primary Somatosensory Cortex

**Figure 3:**
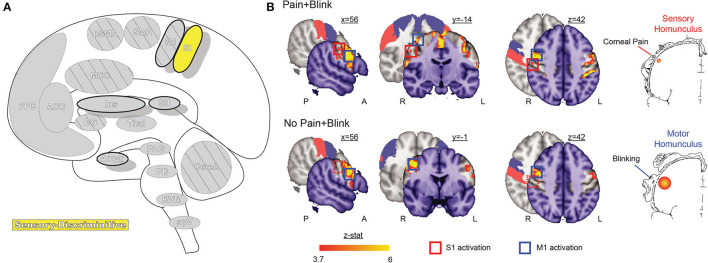
Primary somatosensory cortex. **(A)** Brain areas active during pain: primary somatosensory cortex (SI) highlighted. **(B)** Functional imaging during exposure to bright light while in a photophobic state results in significant activation along the rostral face portion of the SI somatotopic map, contralateral to the site of corneal abrasion. SI activity is no longer present after symptoms resolve, while bilateral M1 and some bilateral SMA activation, associated with blinking, is seen in both conditions. Reprinted from PLOS ONE, Vol 7(9), EA Moulton, L Becerra, P Rosenthal, D Borsook, An approach to localizing corneal pain representation in human primary somatosensory cortex, e4463, Copyright (2012) Moulton et al., under the Creative Commons Attribution License (CC-BY). DOI: https://doi.org/10.1371/journal.pone.0044643SI, primary somatosensory cortex; M1, primary motor cortex; A, anterior; L, left; P, posterior; R, right.

The primary somatosensory cortex (SI, Figure 3) receives input from multiple thalamic nuclei, including lateral thalamic regions associated with processing sensory-discriminative aspects of noxious stimulation. SI is involved in multiple aspects of sensory encoding and integration, from non-noxious heat, proprioception, pressure, type and quality of touch to painful nociception (64–67). Beyond the thalamic connections, SI has dense cortico-cortical connections to multiple other areas, especially the secondary somatosensory cortex and insula, as well as other sensory regions, such as the visual cortex (68–70). The varied and often direct interconnections between these regions and SI are thought to support the role of SI in multisensory integration and actions (69–71). SI is divided into four subregions—Brodmann Areas 3a, 3b, 1, and 2—each suspected of containing a separate mirrored somatotopic map (72, 73). Areas 3a and 3b form one functional parcellation of SI, while areas 1 and 2 form the other; differences in connectivity to thalamic nuclei, other SI subregions, and multiple cortical areas, including motor and frontal cortex, suggest further divisions of function that remain to be explored (74).

Like the TBNC, the face representations in SI are somatotopically organized in an “onion-skin or dermatomal” model, wherein rostral areas are represented inferior and lateral to caudal areas (44, 68, 75). Although the representation of the eye has not been extensively mapped, an fMRI case study localized corneal pain within the rostral-most representation of the face (76).

SI nociceptive responses have been related to the sensory-discriminative aspect of pain, specifically the quality, location, and intensity of stimulus contralateral to the side in which cortical activation is observed, as described by many neuroimaging and lesion studies (64, 66, 77–79). These findings are consistent with direct electrophysiological recordings in primates (65, 67, 80–82).

Research into SI in multiple forms of pain, hyperalgesia, and allodynia describe not only functional changes but related structural changes, including somatotopic reorganization and altered gray matter (75, 83–89). These altered patterns of gray matter density and BOLD signal in the somatosensory cortex are often found during investigations of trigeminal neuropathy and chronic pain (90–93). The changes in neuron excitability, inhibition, or synaptic transmission in the primary somatosensory cortex can affect the perception of pain by its influences on other connected cortical and limbic areas as well as subsequent altered interpretation of peripheral input (16, 94–97).

Recent investigations into the expression of pain on the face have found another relationship between SI and pain (98). The facial expression of pain can be measured for clinical and research purposes, and one method of quantification is through the Facial Action Coding System (FACS) in which non-verbal pain communications are described in Action Units (AUs). AUs are a small set of facial movements shown to consistently occur during pain that can include opening of the mouth and constriction of the muscles around the eyes, among others (99, 100). While AUs are highly variable between individuals in pain, the contraction of the orbicularis oculi muscle surrounding the eyes, a specific AU, is found consistently across subjects and in both acute and chronic pain (99). The sensory-discriminative component of pain is closely associated with orbicularis oculi contraction AU (101), while other AUs are linked to the affective component. The orbicularis oculi AU is mirrored by SI activations that correspond somatotopically to the site of painful stimulus and may serve a protective role by narrowing the eye aperture to shield the eye while preserving vision in dangerous and painful conditions (98). Pain affect-associated AUs are largely thought to be involved in communicating pain to others. Coordinated muscle contractions correspond to SI activity, and contribute toward SI responses observed with ocular pain (98).

Despite many investigations, the role of SI in pain is not fully understood, as activation is not consistently seen across many neuroimaging meta-analyses (7, 8, 10). Focal SI lesions in patients transiently decrease pain sensitivity (102, 103), and direct electrode stimulation of SI does not elicit pain (104). Increasingly, SI is viewed as an area for signal integration from multiple afferent sources, with the diverse classes of fiber inputs combining their transmissions and modifying them intracortically. The resulting signal may be greater or less than expected due to anatomical variability between subjects, non-noxious peripheral inputs, cognitive and attentional factors, and mixed excitatory and inhibitory processes (7, 10, 67, 105).

### Secondary Somatosensory Cortex

**Figure 4:**
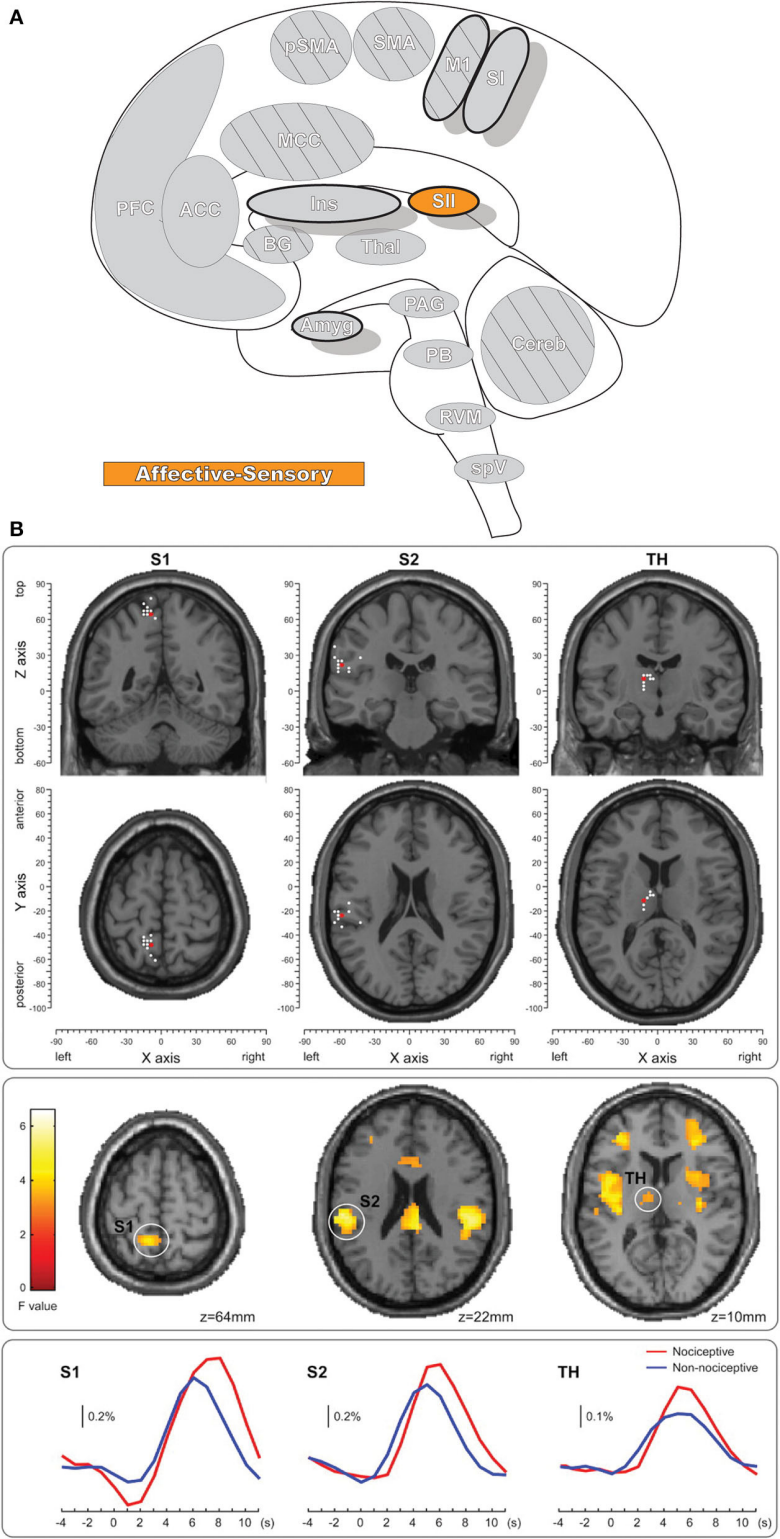
Secondary somatosensory cortex. **(A)** Brain areas active during pain: secondary somatosensory cortex (SII) highlighted. **(B)** fMRI recordings during nociceptive and non-nociceptive stimulation in SI, SII, and Thalamus. The consistent time courses across all three regions suggest parallel information processing in the primary and secondary somatosensory cortices, along with associated activations in the thalamus. Reprinted from The Journal of Neuroscience, Vol 31(24), M Liang, A Mouraux, GD Iannetti, Parallel processing of nociceptive and non-nociceptive somatosensory information in the human primary and secondary somatosensory cortices: evidence from dynamic causal modeling of functional magnetic resonance imaging data, p8976–85, Copyright (2011) Liang et al., under the Attribution-Non Commercial-Share Alike 3.0 Unported License (CC BY-NC-SA). DOI: https://doi.org/10.1523/JNEUROSCI.6207-10.2011white dots, activation maxima for each subject within a given region; red dots, activation maxima across the group within a given region.

The secondary somatosensory cortex (SII, Figure 4) receives nociceptive and innocuous somatosensory information from the thalamus simultaneously by separate but parallel neuronal connections to the pathway leading from thalamus to the SI (106–108). The region also has significant connectivity with the inferior parietal cortex and SI as well as other connections with the intraparietal sulcus, Broca's region, primary motor cortex, and pre-motor cortex (109). SII is more frequently activated than SI in response to noxious stimuli and is one of the most consistently activated brain regions to painful stimuli, along with the thalamus, medial cingulate cortex (MCC), and insula (7, 8, 110). Like SI, SII is involved in the processing of nociceptive afferent input in humans, and likewise has a role in the sensory-descriptive aspect of pain in the lateral pain system (7, 8, 104, 111–113). SII has reduced spatial resolution and receptive field size when compared with SI; unlike SI it has a role in processing other, “high-order” aspects of stimulus including attention, learning, memory, and rare or novel stimuli (113–115). SII is further activated when observing others in physical pain, and even in social-rejection related distress (116, 117). While SI activity is closely associated with the intensity of pain, SII activity is minimal for low-intensity thermal stimuli and increases quickly after exposure to high-intensity stimuli (118). However, note that both SI and SII also respond to pleasant brushing (119) and innocuous heat (105), indicating that activity in these regions is not specific to pain.

SII is frequently divided into four subregions that are loosely homologous to primate areas, termed OP1 (S2); OP2 (parietoinsular vestibular cortex); OP3 (ventral somatosensory area); and OP4 (parietal ventral area) (OP = operculum parietale) (109, 120, 121). Nomenclature of these areas can lack consistency and clarity; notably OP1 is often termed “S2” or “area SII,” leading to some confusion in the literature between the subregion and the overall SII (109). Of these regions, OP1 and OP4 are the most widely studied and are considered somatosensory areas; both subregions contain a complete somatotopic map mirrored along the anatomical border separating them (122). OP1 is considered an integrative area that may facilitate higher-order complex somatosensory processing, while OP4 has greater associations with action control and sensory-motor integration (109).

Activation in SII is bilateral, and this activity increases as the stimulus intensity becomes more painful, which may include engagement of additional SII subregions (113, 118, 123–125). The bilateral activation of SII is non-symmetrical and shows greater activation contralaterally, compared to ipsilaterally (124, 126). This difference reflects the non-equal inputs to the ipsilateral and contralateral SII—contralateral SII is innervated by thalamic nuclei and SI, while ipsilateral SII receives input from contralateral SII and ipsilateral thalamic nuclei (124, 126–128). SII is implicated in identifying, discriminating between, and directing attention to stimuli, cognitively recognizing the painful nature of nociceptive activation, and integrating it with higher-level processes such as learning and memory (61, 109, 115, 118). Some evidence in experiments involving painful stimuli further suggest SII plays a role in processing pain-related emotion that may also include the detection and storage of emotion-laden information regarding potentially damaging stimuli (61). Abnormal pain processing as well as functional and anatomical changes are found in SII in a variety of painful conditions, and SII may be a target for future interventions (129–133).

### Cingulate Cortex

**Figure 5:**
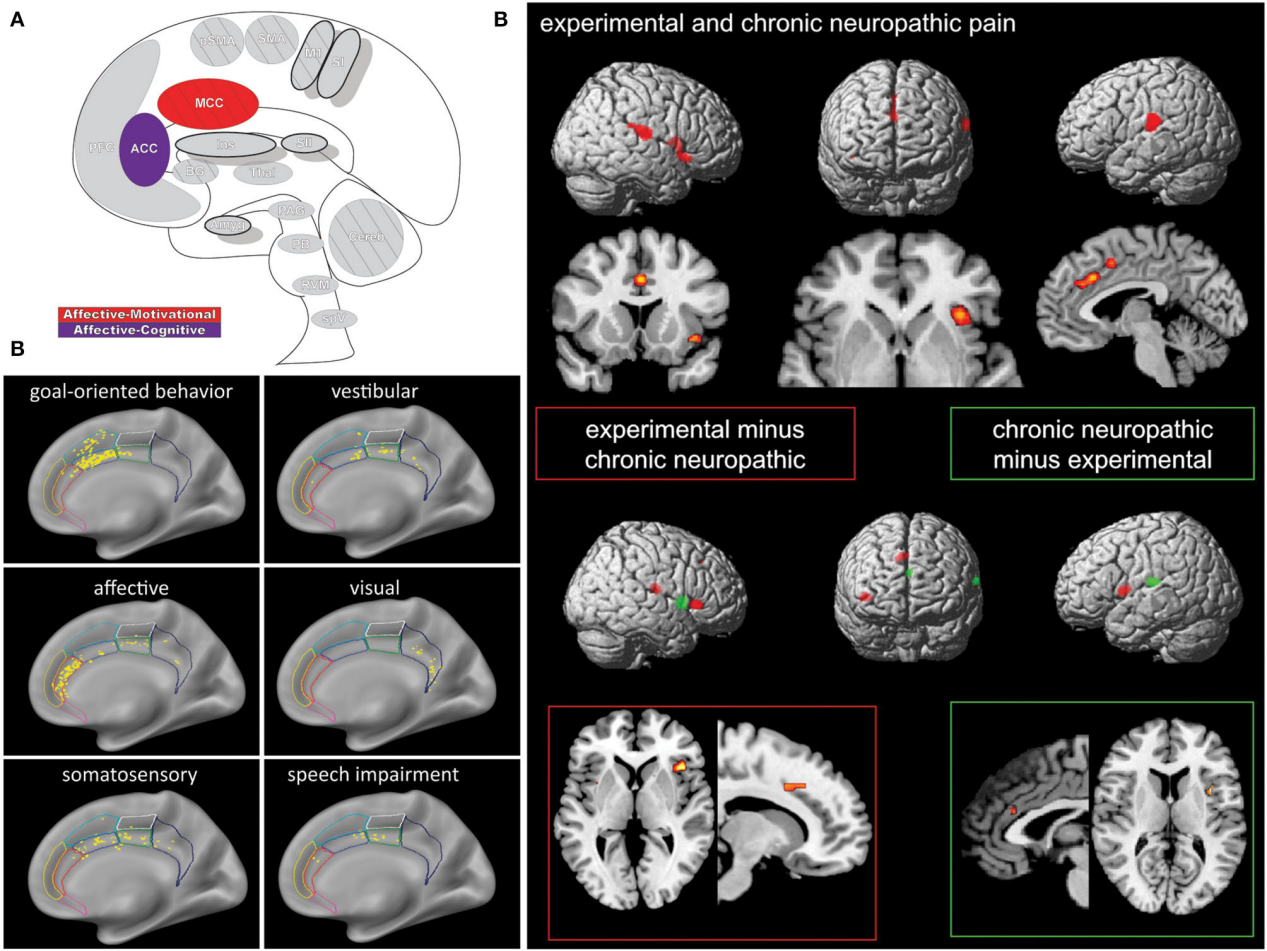
Cingulate cortex. **(A)** Brain areas active during pain: midcingulate cortex (MCC) and anterior cingulate cortex (ACC) highlighted. **(B)** High frequency electrode stimulation across 1789 cingulate sites can elicit varying subjective and behavioral responses segregated into functional fields organized rostrocaudally along the cingulum. F Caruana, M Gerbella, P Avanzini, F Gozzo, V Pelliccia, R Mai, RO Abdollahi, F Cardinale, I Sartori, GL Russo, G Rizzolatti, Motor and emotional behaviours elicited by electrical stimulation of the human cingulate cortex, Brain, Copyright (2018), Vol 141(10), p3035–3051, by permission of Oxford University Press. DOI: https://doi.org/10.1093/brain/awy219. **(C)** Conjunction (top panel) and contrast (bottom panels) analyses of brain regions activated during chronic neuropathic and experimental pain reveal different patterns of activation, implicating several regions as potential actors in chronic pain- including the ACC. Conjunction analysis of both conditions showed activations in the ACC, MCC, SII, insula, thalamus, and supplementary motor area. Experimental - chronic neuropathic pain analysis (red box) resulted in activations in the MCC, anterior and posterior insula, and SMA. Chronic neuropathic - experimental pain (green box) revealed significant ACC, SII, and mid insular activations. Reprinted from NeuroImage, Vol 58(4), U Friebel, SB Eickhoff, M Lotze, Coordinate-based meta-analysis of experimentally induced and chronic persistent neuropathic pain, p1070–80, Copyright (2011), with permission from Elsevier. DOI: https://doi.org/10.1016/j.neuroimage.2011.07.022.

The modern view of the cingulate cortex is a four-region model composed of the Anterior-, Mid-, and Posterior Cingulate Cortex (ACC, MCC, PCC) and the Retrosplenial Cortex (RSC), based on synaptic and functional differences in both primates and humans (134–136). Functional imaging studies in pain have helped affirm the existence of the MCC as a separate functional region rather than a transition area or subsection of the ACC or PCC. Taken together the cingulum as a whole participates in a broad array of somatosensory, emotional, and motor processes, however, fMRI recordings of painful stimuli find consistent activations in the MCC more so than other areas of the cingulum (7, 10, 136–138) (Figure 5).

#### Mid-cingulate Cortex

MCC receives projections from medial and intralaminar thalamic nuclei, including it in the medial pain system, but is also connected to other cingulate regions as well as the insula, amygdala, parietal cortex, striatum, spinal cord, motor, and pre-motor cortices, and many of these pathways are reciprocal (135, 139, 140). The MCC is further divided into anterior (aMCC) and posterior (pMCC) regions (136, 141) and partly contains two of the three cingulate motor zones (or premotor areas) (142). The anterior rostral cingulate zone (RCZa) and the posterior rostral cingulate zone (RCZp) are both somatotopically organized, containing face- and eye-related fields as well as limb motor representations (142). These premotor areas are heavily connected to other brain motor centers, are involved in coordinated emotionally charged or context-dependent movements, such as rubbing or wincing, and are active in a variety of reward and innocuous nociceptive stimuli responses in addition to painful ones (66, 136, 139, 143, 144).

The RCZa is likely within the aMCC and displays strong functional connectivity with the prefrontal cortex, implicating the involvement of cognitive processes (145, 146). The aMCC receives relatively more medial thalamic nuclei innervation than the pMCC as well as a direct input from the amygdala and is active during fear (135, 139). Further functional and anatomical connections arise from the primary motor cortex and insula, and primate studies reveal other connections to the periaqueductal gray and spinothalamic system (145, 147). The same sites in the aMCC are activated by pain and itch and are also involved in dopaminergic reward systems (136, 139, 148). Further, activation is found in the aMCC in the expectation of pain and itch relief as well as pain empathy (136). Functional activity during pain, cognitive control, negative affect, and motor control all overlap in the aMCC, implicating it as being involved in sensorimotor integration that subsequently guides behavior (145, 147). In the context of pain, the aMCC can cognitively assess, experience, and anticipate pain and integrate negative affect into its output (136, 139, 147). The sensorimotor integration allows for a premotor signal that alters behavior and motor response selection based on context provided by numerous systems, with pain resulting in enhancement of specific avoidance and nocifensive motor actions (136, 139). The aMCC is also involved in monitoring the resulting action triggered by its pre-motor signal, sustaining it, and the reward coding of the selected behavior, participating in feedback-mediated decision making (136, 149, 150). Fear can produce many of the same movement activities as pain in the aMCC, and has a similar dynamic in autonomic areas of the ACC, which has led to the classification of fear as a premotor pain signal by some (148).

Conversely, activation in the RCZp in the pMCC is strongly associated with that in the motor cortex and more weakly with the prefrontal cortex (136, 146). The pMCC has more extensive input from the parietal lobe than the aMCC does but no connections to the amygdala and almost no activation in emotion studies (136, 139). Scratching an itch and orienting the eyes to focus on potentially noxious visual targets both show activation in pMCC, and more severe stimuli, or threat of stimuli, result in larger responses (66, 136). Multisensory information from the parietal connections is used by the pMCC to capture attention, guide quick and precise reflexive movements, and orient the body toward impending or realized external multisensory stimuli, including painful ones (136, 139, 140, 144).

#### Anterior Cingulate Cortex

The anterior cingulate cortex (ACC) stores emotionally-valenced memory, has a role in autonomic processes, and serves to integrate these two functionalities (139). The ACC receives medial thalamic innervation, although less so than the aMCC, the orbitofrontal cortex, amygdala, and parahippocampal gyrus (42, 139, 151). Through its OFC connections and downstream, descending pain modulatory sites, the ACC has also been implicated in pain inhibition and facilitation (151, 152). Pain relief from intervention in the ACC usually manifests as a reduction in the perceived unpleasantness or associated distress, highlighting its role in the medial pain system and affect (153). The ACC is often parcellated as two areas: the pregenual (pACC) and the subgenual (sACC) (148, 154, 155).

Activity in pACC is associated with happiness and related memories are stored there (135, 148). Activation in the region by positive memories and events represent reward values that are related to experienced pleasure and show robust functional connectivity to areas of the medial orbitofrontal cortex with similar positive associations and reward (148, 155). The pACC has projections to the facial region of the motor nucleus and is heavily involved in emotion and internal state expression through these projections and the anterior rostral cingulate zone (135, 139). Emotional awareness, common value scaling, and cost assessment are also functions carried out by the pACC, and the subregion can help make decisions involving reward/punishment tradeoffs (135, 139, 148). Opioid receptors are dense in the ACC, and the application of naloxone negates activity in the pACC during nociception, showing the key role of this area in antinociceptive processes (156).

sACC activity is maximal during negatively valenced stimuli and events, and the subregion stores memories associated with sadness (139, 148, 157). Fear also results in notable activation of the sACC (148). Like the pACC, the sACC receives OFC inputs but from the punishment-related and negatively associated lateral orbitofrontal cortex (148, 151). The sACC is strongly connected to the amygdala, lateral hypothalamus, PAG, and parabrachial nucleus (139, 148), and these outputs underscore the role this region plays as an integrative autonomic center (139, 148, 158). Enhanced sACC activity is found in numerous pain studies and is associated with reduced pain; diversion, placebo, habituation, pain adaptation, expectancy, and reward all seem to function through the activation of brainstem descending pain pathways initiated by the ACC (148, 151, 155).

ACC is activated in pain neuroimaging experiments far less frequently than the MCC, likely due to the fact that ACC activation is seen when the stimulus or pain is intense enough to engage descending pain control systems (152). Significant confusion surrounds cingulate pain neuroimaging, often due to the evolving subregional nomenclature (famously, dACC is not the same as aMCC), and the work of many meta-analyses has been devoted to reclassifying data to fit the new models (136, 148). Thus, over time, the ACC has “lost” some of its presumed functioning in pain as those nociceptive activations are correctly reassigned to other cingulate regions (148).

### Insular Cortex

**Figure 6:**
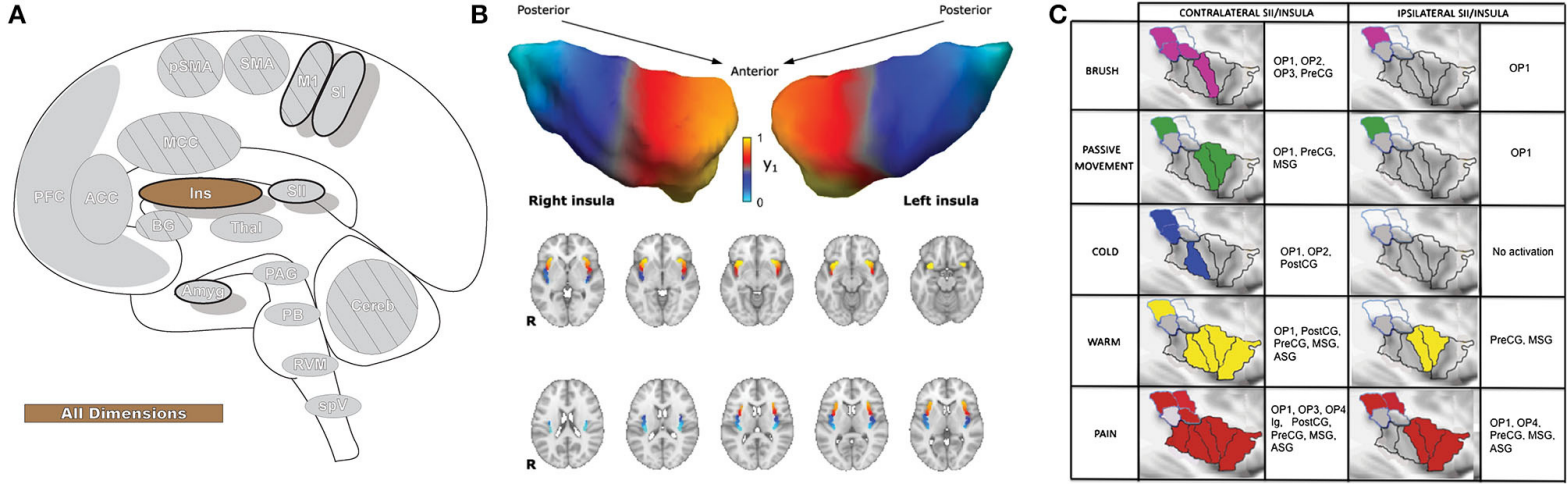
Insular cortex. **(A)** Brain areas active during pain: insula (Ins) highlighted. **(B)** Topographic organization of connectivity (anatomical and FC) of the insula and other brain regions is arranged along a rostro-caudal gradient wherein anterior insular regions show strong connections to the anterior cingulate cortex, dorsolateral prefrontal cortex, and inferior parietal lobules (red) and the posterior insula with somatosensory regions and the parietal operculum (blue). Similarities in connectivity profiles in adjacent insular regions suggest that, rather than discrete subunits, the topographic distribution of connections is better appreciated as a spatially continuous and gradually changing gradient. Displayed as a gradient in graph form, this type of spatial connectivity analysis is referred to as a connectopy map. FC,functional connectivity- temporally synchronized low-frequency fluctuations in BOLD signal between regions that indicate they are connected in their functions. Such areas may or may not have direct anatomical connections. Reprinted from Nature: Scientific Reports, Vol 22(1), D Vereb, B Kincses, T Spisak, F Schlitt, N Szabo, P Farago, K Kocsis, B Bozsik, E Toth, A Kiraly, M Zunhammer, T Schmidt-Wilcke, U Bingel, ZT Kincses, Resting-state functional heterogeneity of the right insula contributes to pain sensitivity, p22945, Copyright (2021) Vereb et al., under the Creative Commons Attribution License (CC-BY). DOI: https://doi.org/10.1038/s41598-021-02474-x. **(C)** Operculo-insular areas (including insula and SII) respond to a wide variety of somatosensory, and painful, stimuli. Anatomically defined region of interest analyses with fMRI indicate varied functional overlap/segregation between a variety of stimuli delivered to the left hand. Reprinted from NeuroImage, Vol 60(1), L Mazzola, I Faillenot, FG Barral, F Mauguiere, R Peyron, Spatial segregation of somato-sensory and pain activations in the human operculo-insular cortex, p409–18., Copyright (2012), with permission from Elsevier. DOI: https://doi.org/10.1016/j.neuroimage.2011.12.072. PreCG,precentral insular gyrus; ASG,anterior short gyrus; MSG,middle short gyrus; posterior- PostCG,postcentral insular gyrus; Ig1,insular lobe granular area 1; Ig2,insular lobe granular layer 2; Id1,insular lobe dysgranular area 1; SII subunits: OP1, OP2, OP3, OP4 (OP, operculum parietale).

The insula (Ins: Figure 6) receives direct nociceptive input from thalamocortical pathways in primates and is a core region activated in essentially all painful experimental conditions, including a wide variety of exteroceptive and interoceptive stimuli (10, 159). The insula is the only brain region that evokes pain when directly stimulated, including pain around the eye (104, 113, 160). The insula is divided into three subregions: the anterior, middle, and posterior; different regions of the insula play a role in sensory, affective, and cognitive aspects of perception (10, 161). Most resources refer to discrete insula subdivisions, and experiments are often designed around this fact. However, recent investigation has suggested the region may be better appreciated as a gradually changing topographical gradient of functional and anatomical connections along the rostrocaudal axis. While discrete subunits are described throughout this manuscript, selecting small/discrete subregions of interest for analysis may provide significant results that may not reflect the totality of activations or connectivity in a given brain region (162, 163).

Anterior insula (AI) is involved in processing emotion, including empathy, and activation of this subregion is found in affective processing (161, 164). AI has strong functional and anatomical connections with the thalamus and cognitive and emotional parts of the prefrontal cortex as well as the amygdala and some cingulate regions, particularly the ACC, and can have increased or decreased functional associations with these areas in chronic pain (163, 164). The strongest connections to the prefrontal cortex are to the dorsolateral prefrontal cortex, as well as areas associated with cognitive-evaluative processing and outcome anticipation (orbitofrontal cortex) and with the regulation of emotions and cognitive pain modulation (ventrolateral prefrontal cortex) (163, 164). Expectation and behavioral avoidance of a negative outcome activate AI, and the region is thought to impose emotional states that are informed by the evaluation of affective events (161, 164). The evaluation of the saliency of various insular inputs, attention, and the engagement of relevant brain regions and their triggered affective and emotional responses (including affective and cognitive pain modulation) are major functions of the AI (10, 161, 164–166). The AI has also been closely tied to autonomic function (167), and neural activity in this area has been correlated with the dynamic magnitude of pupillary dilation (168).

The mid insular (MI) area has connections to SI and SII (sensory-discriminative) as well as the ventrolateral prefrontal cortex (affective-emotional-cognitive), and diverse outputs to the orbitofrontal and premotor cortices, parietal and temporal brain regions, and the inferior frontal gyrus (164). Based on the diversity of input and output, the MI is viewed as a hybrid medial/lateral pain system area that integrates the diverse components of pain (164).

Posterior insular (PI) regions are thought to process interoceptive, somatosensory, visceral, and pain stimuli (7, 161, 164, 165). PI has its strongest connections to SII (structural and resting state analyses) and SI (structural) along with other somatosensory areas, and has some resting state association with the pMCC as well (163, 164). Activations in PI are found as stimuli progress from innocuous to painful in intensity with little activation in the absence of noxious input. Thalamic nuclei, insula, SI, and SII together comprise the lateral pain system and sensory-discriminative pain; however, lateral thalamic nuclei have been shown to have low connection probability to PI, in contrast to many primate tracing studies.

While most other cortical areas are activated by distinct components of pain, insula appears to have sub-regions dedicated to processing and integrating a wide array of intero- and exteroceptive information as well as the focusing of cognitive and perceptive attention to the most salient of these inputs (161, 164–166, 169). Insular lesions can result in pain asymbolia, wherein patients recognize the presence of pain but are devoid of proper emotional and motor responses and may not react to visual or auditory threats (170). The inappropriate reaction to pain caused by insular damage highlights the importance of the region in serving to join the sensory and limbic systems and correctly process and integrate the affective-motivational component of pain with the other dimensions (170). While multiple studies have looked into the role of the insula in pain, as indeed it is the most consistently activated region in pain-related neuroimaging studies (7, 10, 171), the insula is also part of a prospective sensory salience network (172). As other regions in the brain have the capacity for multimodal sensory integration and can be active during painful stimulation, the question remains as to whether activations in insula truly reflect the various dimensions of pain or whether they process saliency and focus attention to particularly salient stimuli (173).

### Amygdala

**Figure 7:**
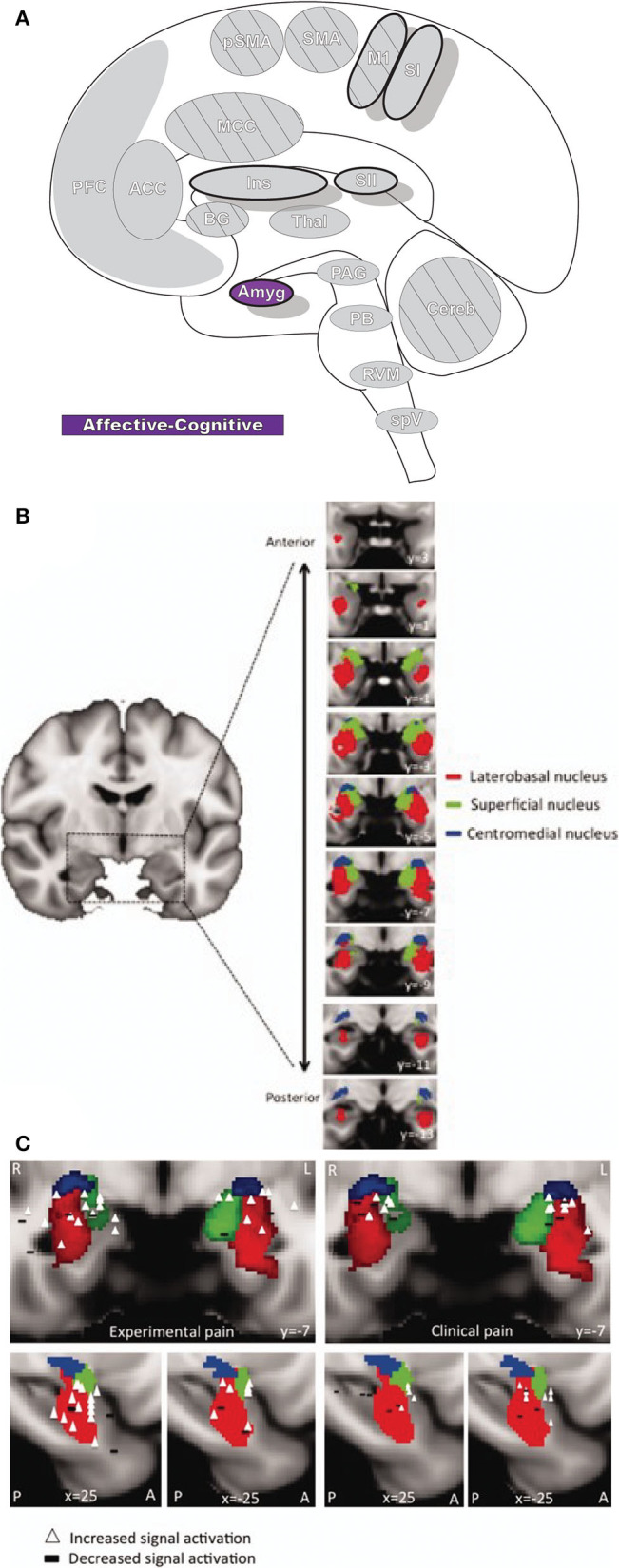
Amygdala. **(A)** Brain areas active during pain: Amygdala (Amyg) highlighted. **(B)** MRI imaging of amygdala subunits displayed in a series of coronal slices. Reprinted from Human Brain Mapping, Vol 35(2), LE Simons, EA Moulton, C Linnman, E Carpino, L Becerra, D Borsook. The human amygdala and pain: Evidence from neuroimaging, p527-38, Copyright (2012), with permission from John Wiley and Sons. DOI: https://doi.org/10.1002/hbm.22199. **(C)** A meta-analysis of functional neuroimaging studies reporting amygdala involvement, including 24 experimental and 17 clinical pain studies, emphasizes the participation of the amygdala in pain. The associations between laterobasal activation and clinical pain, as well as the centromedial/superficial regions and experimental pain, support previously reported anatomic and functional parcellations of the amygdala. White triangles,increased signal activation vs controls reported; black dashes,decreased signal activation vs controls reported. Reprinted from Human Brain Mapping, Vol 35(2), LE Simons, EA Moulton, C Linnman, E Carpino, L Becerra, D Borsook et al., The human amygdala and pain: Evidence from neuroimaging, p527–38, Copyright (2012), with permission from John Wiley and Sons. DOI: https://doi.org/10.1002/hbm.22199.

The amygdala (Amyg: Figure 7) is directly involved in emotional processing (174) and has a major role in aversive, fear-based learning and negative affect, as well as motivation and reward learning (175–178). The amygdala is a highly interconnected region of the brain, with dense afferent and efferent connections extending widely (178, 179). Nuclei of the amygdala are typically divided into superficial, laterobasal, and centromedial groupings based on cytoarchitecture and diffusion-tensor imaging studies (180–182). The superficial division is largely concerned with olfactory processes; however, some studies have found functional connectivity associations with other limbic regions that imply a potentially larger role in affect (178, 183). The laterobasal and centromedial groups are important for the transmission of nociceptive signals and, through their diverse connections to other brain regions, are implicated in emotional and affective-motivational components of pain, as well as the cognitive-evaluative dimension (178).

The laterobasal division has extensive innervation from numerous modalities, including nociceptive input *via* the somatosensory thalamus and multiple cortical and subcortical areas, such as hippocampus, ACC, and insula (184, 185). Laterobasal amygdala is involved in associative learning, as with fear-based classical conditioning, thereby giving sensory information emotional significance, and as such is important in anxiety and fear related to pain (178, 186). Additionally, the laterobasal nuclei group has connections with parts of the striatum as well as prefrontal and frontal cortices, which contribute to pain memory and expectation, important parts of the cognitive-evaluative component of pain and the anticipation of pain (178, 187, 188).

The amygdala's centromedial nuclei are a major target of excitatory and inhibitory sensory input from other amygdala nuclei groups and they also receive nociceptive information from the medullary dorsal horn, cingulate cortex, and insula as well as the lateral parabrachial nucleus complex (47, 179). This information is projected to the nearby bed nucleus of the stria terminalis, hypothalamus, PAG, striatum, and several other brainstem regions (178, 179, 184). The connectivity between the amygdala and these areas underlies its significance in generating behavioral responses to painful stimuli as well as modulating the subsequent emotional, autonomic, behavioral, and endocrine pain responses (178, 185).

### Prefrontal Cortex

**Figure 8:**
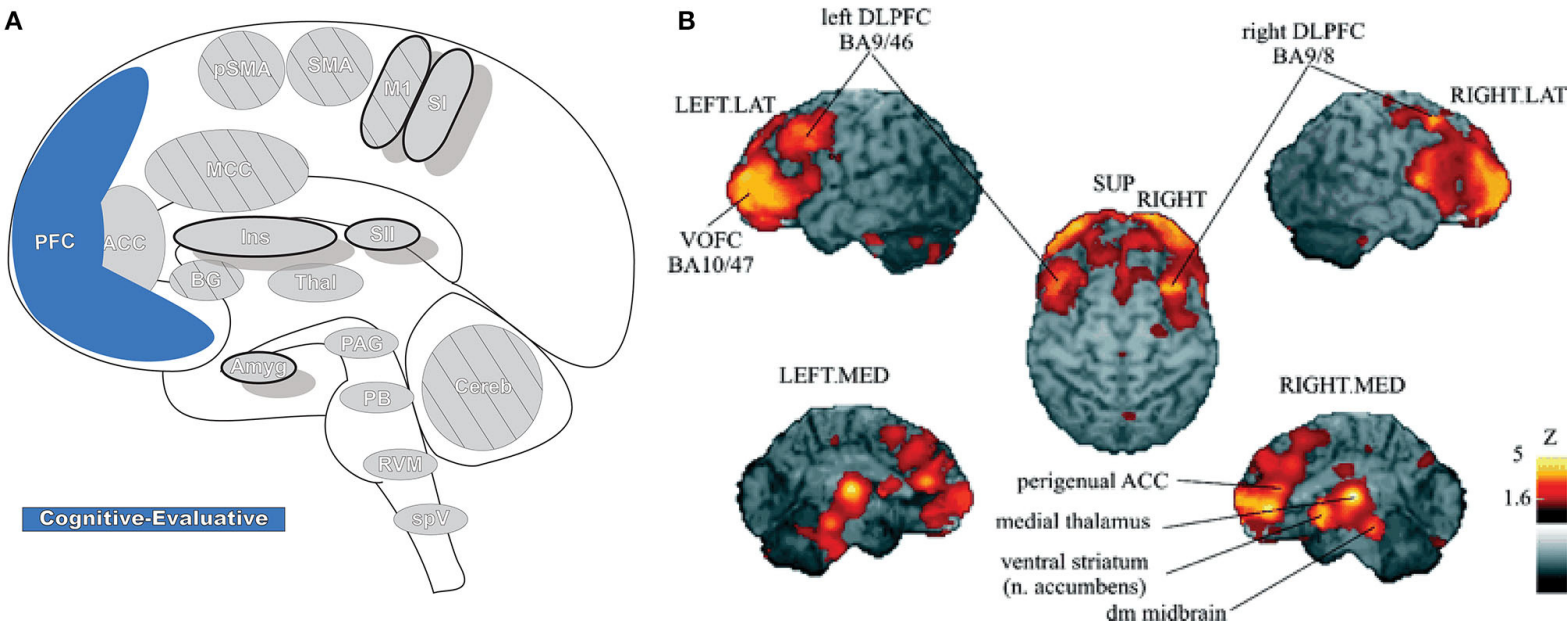
Prefrontal cortex. **(A)** Brain areas active during pain: prefrontal cortex (PFC) highlighted. **(B)** Enhanced activation of ventral/orbitofrontal cortex (VOFC) and dorsolateral prefrontal cortex (DLPFC) during experimentally induced heat allodynia compared to equally intense heat pain stimuli. This difference demonstrates the nuanced response of the PFC in pain processing in different contexts. The basal ganglia were also found significantly more active in allodynia. Reprinted from J Lorenz, S Minoshima, KL Casey, Keeping pain out of mind: the role of the dorsolateral prefrontal cortex in pain modulation, Brain, Copyright (2003), Vol 126(5), p1079–91, by permission of Oxford University Press. DOI: https://doi.org/10.1093/brain/awg102. VOFC, ventral/orbitofrontal cortex; DLPFC, dorsolateral prefrontal cortex; LAT, lateral; MED, media; SUP, superior; dm, dorsomedial.

Prefrontal cortex (PFC: Figure 8) is critical for cognitive control (the manipulation of information in pursuit of a goal) and can represent abstract information and complex rules that subsequently inform thoughts, emotions, and actions. It participates in high-order, intelligent planning and problem solving, emotion generation and regulation, and other executive functions (189–191). PFC is believed to be organized in a hierarchal rostro-caudal axis, in which posterior areas are involved in control of short-term and concrete action representations while complex, longer-term representations occur in progressively more anterior areas as information and control selection become increasingly abstract (146, 189, 192). Painful stimuli can engage many of these high-order processes, and multiple areas of PFC are involved in pain processing (190). In PFC, nociceptive signals are gathered with other contextual information (e.g., memories and emotions) into a unified, processed perception that then modulates peripheral nociception by its projections to the PAG (190).

The parcellation and nomenclature of PFC subregions is not consistent in the literature; cytoarchitectonic areas (e.g., Brodmann) can be assigned to different subregions depending on the study. Likewise, some subregion divisions can include functionally and anatomically distinct areas that may be referred to differently between studies and across disciplines (191, 193). A unified systematic nomenclature and parcellation may help ease the difficulties in investigating large-scale, integrative PFC functionality (191). The challenges and shortcomings in nomenclature are not unique to PFC—as neuroimaging techniques become more sophisticated it seems clear that establishing common representations and analysis methodologies throughout the brain can serve to advance the field (194–196). In this review, PFC is divided into orbitofrontal cortex (OFC), medial prefrontal cortex (MPFC), lateral prefrontal cortex (LPFC), and anterior prefrontal cortex (APFC), each with its own internal functional parcellations; the pACC, sACC, and aMCC are also often considered functionally to be part of the PFC (191).

LPFC activity is found frequently in neuroimaging of pain and cognitive control, and the subregion can be divided into dorsolateral and ventrolateral prefrontal cortex (DLPFC, VLPFC) (10, 189, 191, 197). DLPFC is also involved in executive function, ranging from attention, decision making, and emotional regulation to working memory and reward/value coding (197). DLPFC is a part of several brain networks, is widely involved in top-down process control and modulation, and has a similar role in the context of pain—cognitive/attentional modulation of pain, reducing emotional pain-responses, placebo analgesia, and other forms of pain suppression (197–199). Many of these phenomena engage circuits involving VLPFC and the ACC, to which DLPFC is interconnected, and are thought to be involved in the initiation of modulatory signaling to downstream effectors in the brainstem (6, 197). Pain detection and spatial discrimination are other implicated functions, as DLPFC activation has been observed in the response to, and anticipation of, painful nociceptive stimulation (190, 197). DLPFC is a site of integration between pain transmission, cognitive expectation, and evaluation of the resultant pain; the results of this processing lead to pain modulation and context-informed behavioral response to painful stimulation (197). Many investigations have found associations between DLPFC activation and enhanced pain in experimentally sensitized nociceptive circuits as well as abnormal anatomical and functional states in chronic pain (190, 197, 198).

VLPFC is innervated by AI, shows activation during pain anticipation, and is functionally associated with cognitive pain control systems (ACC, PAG, and RVM) (198, 200). VLPFC also shows increased activity in painful stimulation with activation often seen during placebo analgesia and other forms of signal manipulation, potentially contributing to pain modulation by reappraisal of the estimated threat an aversive stimulus represents (198, 201, 202). The delineation and initiation of functional process between the LPFCs by neuroimaging is complicated by close connections between the two regions (DLPFC and VLPFC), their dual involvement in expectation and emotion-regulation, and shared inverse association between pain-expectant cognitive processes and catastrophizing (201). Likewise, both DLPFC and VLPFC have been found to function abnormally in some cases of chronic pain (190, 198, 201).

MPFC, often divided into dorsomedial and ventromedial subregions, has a prominent role in aversive learning, processes the affective and cognitive components of pain, and functionally includes portions of the ACC and MCC (191, 203). Considered together, projections in primate tracings and supporting fMRI functional studies have found connections from MPFC to the PAG, comprising a large portion of the overall input to the critical pain modulatory region (190). Additional tracts are seen between the MPFC, amygdala, thalamus, hypothalamus, and rostral ventromedial medulla, providing a potential way that emotion (i.e., fear) can influence pain modulation as well as function in empathy toward pain or suffering (190). Cognitive inhibition of emotional and pain responses, including motor and facial expression such as orbicularis oculi contraction, is thought to be learned and can occur through coordinated activations in the MPFC, basal ganglia, and cingulate regions that can be disrupted as the perception or suggested intensity of sensory-discriminative pain increases (98, 191). MPFC connections to parietal areas underlie the processing of emotionally-valenced visual stimuli that affect associated nociceptive signaling, a form of cognitive pain modulation (190).

OFC, containing medial and lateral subdivisions, has connections to many pain-processing areas, including the insula, ACC, and somatosensory cortex, and shows increased activation during exposure to uncontrollable and unpredictable pain and its accompanying sensitization as well as to the fear of pain (191, 202, 204). OFC processes negative and punishment-related aspects of stimuli as well as the context-dependent value of a reward (204, 205). In the presence of both pain and reward, functional coupling between OFC and other cerebral pain centers is disrupted, resulting in higher-order signal modulation and pain inhibition (205). Like other parts of PFC, many studies have revealed alterations of this area in chronic pain, although OFC changes may not only reflect the modulation of nociceptive signaling but instead interactions between pain and reward (190, 205).

APFC, like other prefrontal regions, has been related to many functions including reward and conflict, working memory, risk and decision making, and pain (206, 207). This area has also been found to be involved in essentially all salient stimuli that may require behavioral response (206). APFC has reciprocal connections to the other PFC subregions, the parietal, insular, and anterior temporal cortices, multiple thalamic nuclei, and numerous other subcortical regions in tracer studies in primates, strongly supported by structural and functional associations in human neuroimaging (206, 207). APFC has a medial and lateral division (mAPFC and lAPFC), which have functionally distinct processes (206, 207). lAPFC in pain is thought to be involved in high-level cognitive sensory and emotional nociceptive signal integration and may modulate pain through its input to the descending antinociceptive circuit (206). On the other hand, mAPFC may have a role in memory-based aversive processing, both past and ongoing, and general stress response while its connections to the medial pain system highlight a potential role in emotional and motivational components of pain (206). Together, APFC regions perform high-level cognitive evaluation of pain, historical and present, and the results of that processing are used to guide behavior (206).

### Primary Motor Area, Supplementary Motor Area, and Pre-supplementary Motor Area

**Figure 9:**
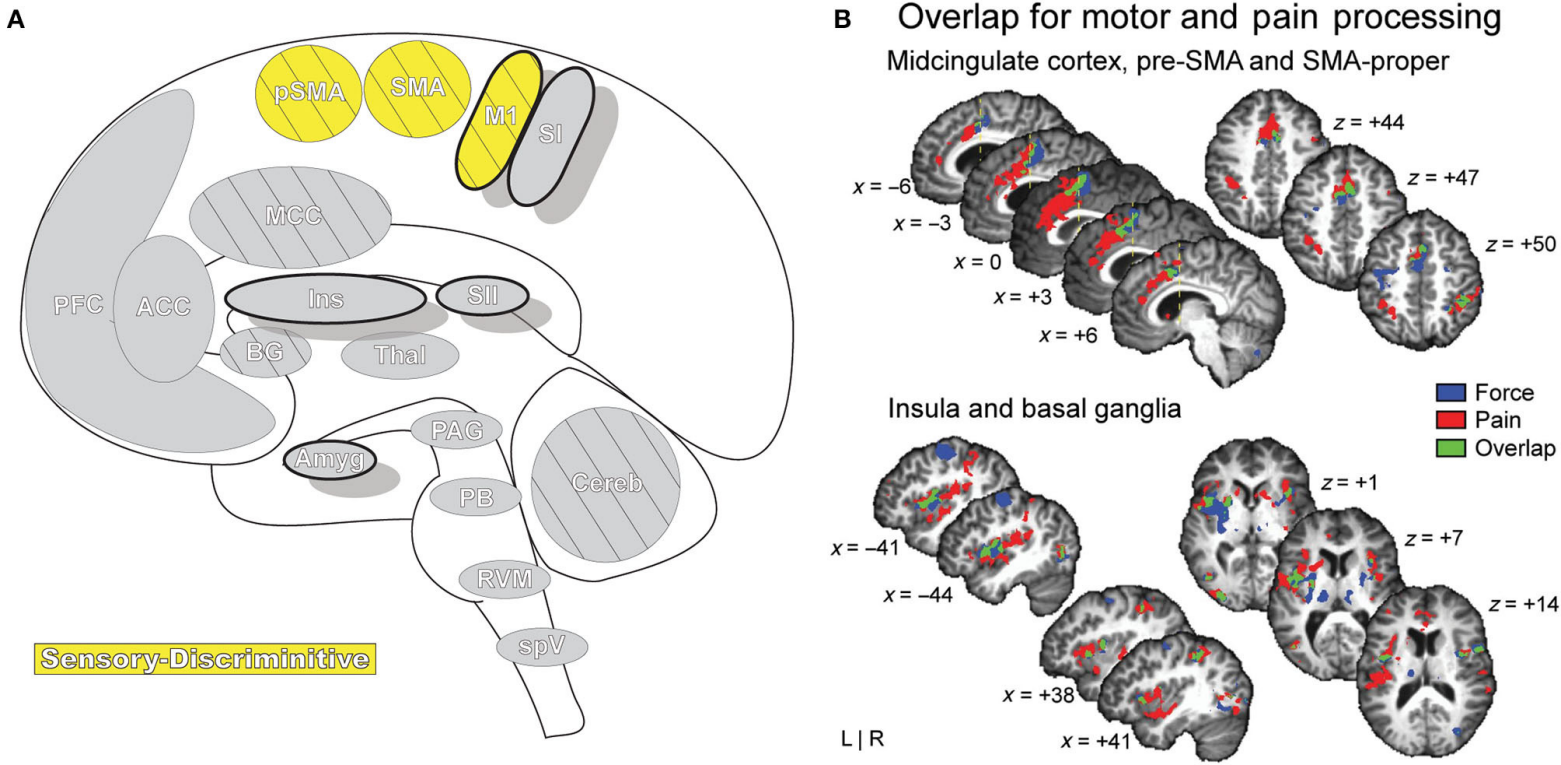
Motor Areas. **(A)** Brain areas active during pain: primary motor area (M1), supplementary motor area (SMA/SMA-proper), pre-supplementary motor area (pSMA/pre-SMA) highlighted. **(B)** Functional activations associated with pain processing (painful heat: red) and motor control (force production: blue) overlap (green) in the SMA, pSMA, and aMCC, and display increased activation when simultaneously processing both conditions. Further results of the same group-level conjunction analysis describe overlap in pain and motor processes in the anterior insula and basal ganglia (putamen), reinforcing a dynamic established previously in the literature. Pain processes were established by painful thermal stimulation to the right hand; motor control processes were established by participants gripping a force transducer with their right hand. Reprinted from G Misra, SA Coombes, Neuroimaging Evidence of Motor Control and Pain Processing in the Human Midcingulate Cortex, Cerebral Cortex, Copyright (2014), Vol 25(7), p1906–19, by permission of Oxford University Press. DOI: https://doi.org/10.1093/cercor/bhu001.

Primary motor cortex (M1), supplementary motor area (SMA), and pre-supplementary motor area (Pre-SMA) are critical areas for the planning and execution of motor output in response to sensory input, and their activation is found in many pain studies (7, 8, 10) (Figure 9). The motor areas have dense innervation between themselves and are heavily connected to the corticospinal tract, parietal cortex, cerebellum, and thalamus (208). The collection of motor and motor planning areas are implicated in behavioral and some reflex responses to pain as well as the anticipation of pain (98, 145, 209).

Pain impacts muscle contraction and coordination and interferes with motor-skill learning (210). Many pain-related motor area activations are thought to be involved in pain-initiated movement or the suppression of pain reflexes and are involved in affective/motivational pain responses (8, 42). The pre-SMA and SMA show activity in similar regions in both pain processing and motor function as well as during the execution of visually guided movement (145). Additionally, emotion can influence motor system responses, and the system may itself code and store emotional context along with motor-processes; activation of the motor system can be seen before and during interaction with an unpleasant stimulus (209, 211). Pain and/or the expectation of pain may alter excitability in motor areas, causing inhibition of certain motor actions, such as further interaction with a negative stimulus; conversely, positively associated stimuli may facilitate motor activities leading to increased interactions (211, 212).

Motor cortex intra- and transcranial stimulation has repeatedly been shown to relieve pain in certain neuropathic conditions, but the mechanism behind this phenomenon is not fully understood (213, 214). Multiple hypotheses exist and may not be exclusionary, involving modulation and regulation of signals to PFC, cingulate cortices, thalamus, brainstem, basal nuclei, and spinal cord (214, 215). The altered excitation of nerve fibers by activation of opioid-releasing structures throughout the brain, active reappraisal of the emotional component of pain, and potential regulation of peripheral feedback imbalances may all contribute to pain relief (214–216).

### Basal Ganglia

**Figure 10:**
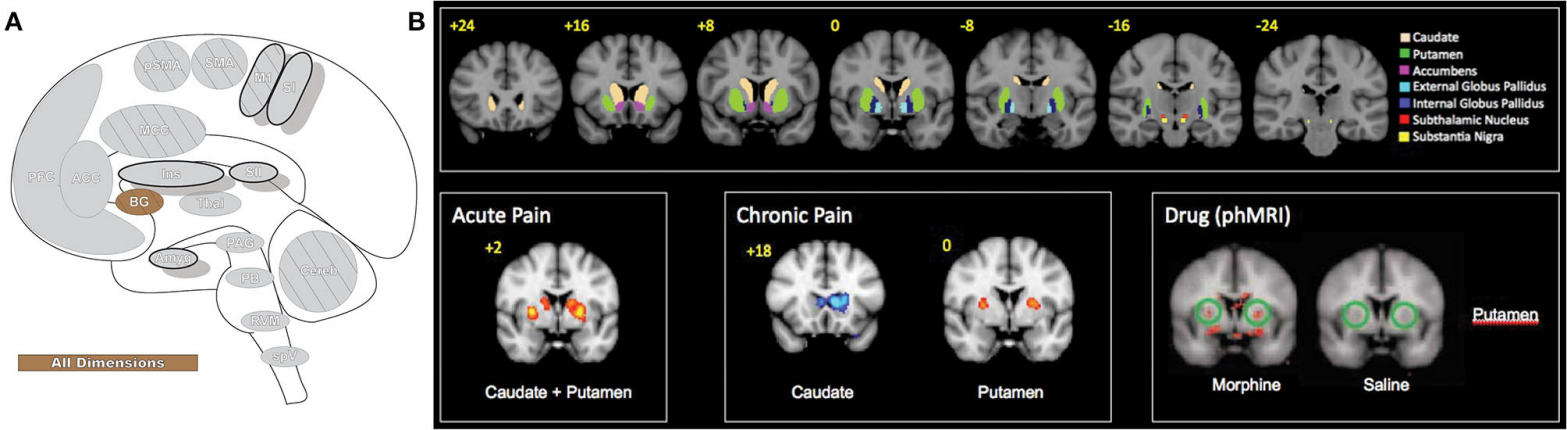
Basal ganglia. **(A)** Brain areas active during pain: basal ganglia (BG) highlighted. **(B)** The basal ganglia participate in pain processing, from acute pain and chronic pain (cold and brush neuropathic allodynia) to morphine-induced analgesia, as revealed by pain-related patterns of fMRI BOLD activity. Upper panels: BG parcellations are color-coded and highlighted in coronal sections organized from anterior to posterior. Bottom panels: red areas indicate increased fMRI BOLD activation and blue areas indicate decreased activation. phMRI, pharmacological MRI- wherein pharmacological agents/drugs (in this case morphine) are used as stimuli to induce hemodynamic changes that are subsequently assessed by fMRI. Reprinted from Molecular Pain, Vol 6(27), D Borsook, J Upadhyay, EH Chudler, L Becerra, A key role of the basal ganglia in pain and analgesia–insights gained through human functional imaging, Copyright (2010) Borsook et al, under the Creative Commons Attribution License (CC-BY). DOI: https://doi.org/10.1186/1744-8069-6-27.

The basal ganglia (BG, Figure 10) are a group of subcortical forebrain nuclei that are highly connected to the cortex, brainstem, and thalamus and are best known for dopaminergic involvement in motor systems and movement control (217–219). BG are involved with planning learned motor behavior execution, directing voluntary movement, and coordinating context-dependent movement (220). BG nuclei include globus pallidus, substantia nigra, striatum, and subthalamic nucleus (219, 221). Striatum is separated into ventral and dorsal subdivisions—the ventral is closely associated with the limbic system and is partially comprised of the nucleus accumbens, while the dorsal striatum consists of caudate nucleus and putamen (219, 221). The striatum is the main input area of the BG and is innervated almost globally by the cortex; these diverse inputs are organized into sensorimotor, cognitive, and affective functional regions with overlap that may reflect integration of cortical information (217, 219). Globus pallidus contains major output nuclei that connect widely to other BG nuclei as well as several thalamic nuclei and midbrain structures (219). One such structure is the superior colliculus, in which BG has a non-looped connection that supports a role in regulating eye movements and behaviors resulting in orientation toward a stimulus (219).

BGs role has expanded beyond movement alone to include cognitive and emotional activity, skill and habit learning, perception, procedural memory, planning, language, and attention (218, 219, 221, 222). This diverse functionality extends to pain processing, where BG are suggested to participate in the affective-motivational, sensory-discriminative and cognitive-evaluative components of pain as well as some analgesic effects and are critical participants in the behavioral resultants of chronic pain (221). Of the BG, the regions most consistently activated in experimental pain the caudate, pallidus, and putamen subregions (10). Caudate is believed to involve pain avoidance behavior and behavioral reinforcement that may include pain (221). Pallidus encodes repertoires of behavior, and deep brain stimulation of this area causes pain inhibition (221). Putamen activates bilaterally while also contralaterally representing somatotopic nociceptive information and potentially playing a role in pain modulation (220, 221). BG have an important role in managing context-dependent movement and, through extensive thalamo-cortical-BG loops, modulate the integration of the diverse components of the pain experience and influence resultant movement behaviors (221).

### Cerebellum

**Figure 11:**
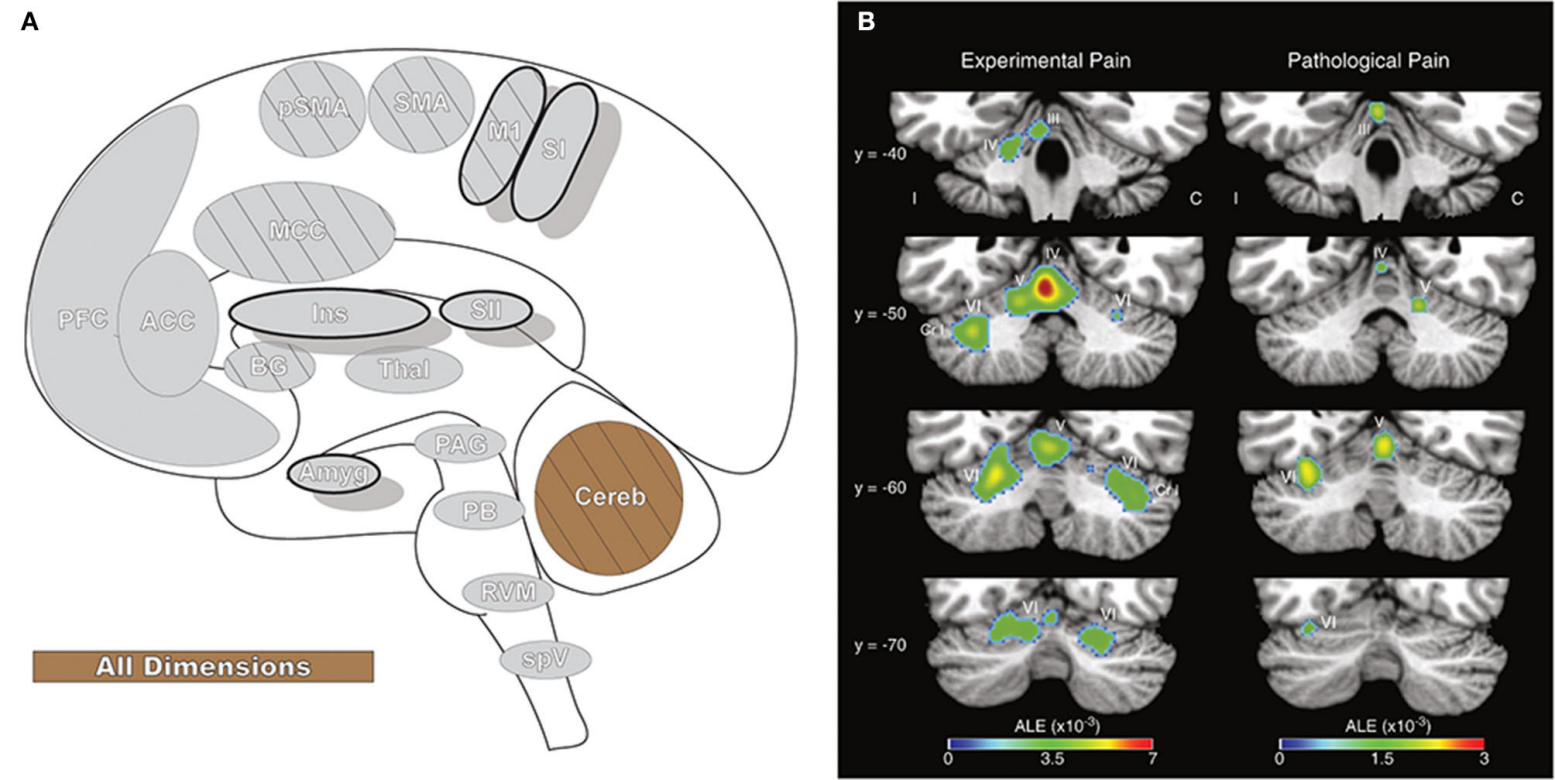
Cerebellum. **(A)** Brain areas active during pain: cerebellum (Cereb) highlighted. **(B)** Cerebellar activation likelihood estimation (ALE), derived from meta-analysis of 56 experimental and 20 pathological pain studies, illustrates that fMRI activity is frequently present in specific cerebellar foci during pain. Reprinted from Brain Research Reviews, Vol 65(1), EA Moulton, JD Schmahmann, L Becerra, D Borsook, The cerebellum and pain: Passive integrator or active participator?, p14–27, Copyright (2010), with permission from Elsevier. DOI: https://doi.org/10.1016/j.brainresrev.2010.05.005. C, activation contralateral to painful stimuli; I, activation ipsilateral to painful stimuli; Cr I, Crus I; III-VI, cerebellar hemispheric lobules III through VI.

The cerebellum (Cereb: Figure 11) is best known for coordinating movement (223). This basic understanding has evolved, as interrogation of the cerebellum has revealed integrative and diverse functionality, from memory and learning to the processing of somatosensory input. The cerebellum has many supraspinal projections that are routed through the brainstem and has reciprocal connections to cortical structures involved in both sensorimotor processing and cognitive functions (224, 225). In addition to supraspinal input, direct afferent pathways pass nociceptive information from peripheral sources through midbrain nuclei to the cerebellum (225, 226). Some evidence suggests a somatotopic organization for these peripheral afferent inputs, while cerebellar regions receiving supraspinal input may be non-somatotopically organized and provide a means for emotional and cognitive information to affect cerebellar sensory-motor processes (227). The cerebellum can be divided into separate functional regions based on anatomic, neuroimaging, and resting state studies. These studies describe anterior cerebellar connections to sensorimotor-related cortical regions, which support motor functions and posterior cerebellar circuits to cognitive and associative cortical regions, which in turn may function in motor planning, nociception or memory (225, 226).

While not observed in all pain neuroimaging studies (10), the cerebellum is often active during pain (7, 8, 226). The cerebellar response to pain is most frequently seen in activation of the anterior vermis and posterior hemispheres, and similar activity in the posterior hemispheres is observed in the neuroimaging of emotion and evocative pictures, particularly those with aversive connotation (226, 228). These same regions may be activated in response to the anticipation of a painful stimulus as well as the stimulus itself (227). Activation of posterior cerebellar regions has been functionally inversely correlated to limbic areas involved in emotional processing (228), and damage to these regions has been linked to disrupted pain affect (229). A positive functional relationship is found between pain and sensorimotor areas, such as M1, SII, AI, and the PAG. The cerebellar pain response may be related to motor planning and reflexes as well as to the activation of a corticocerebellar aversive network that modulates sensitivity to negative events by connectivity to cognitive and emotional brain regions (225, 228).

### Brainstem

**Figure 12:**
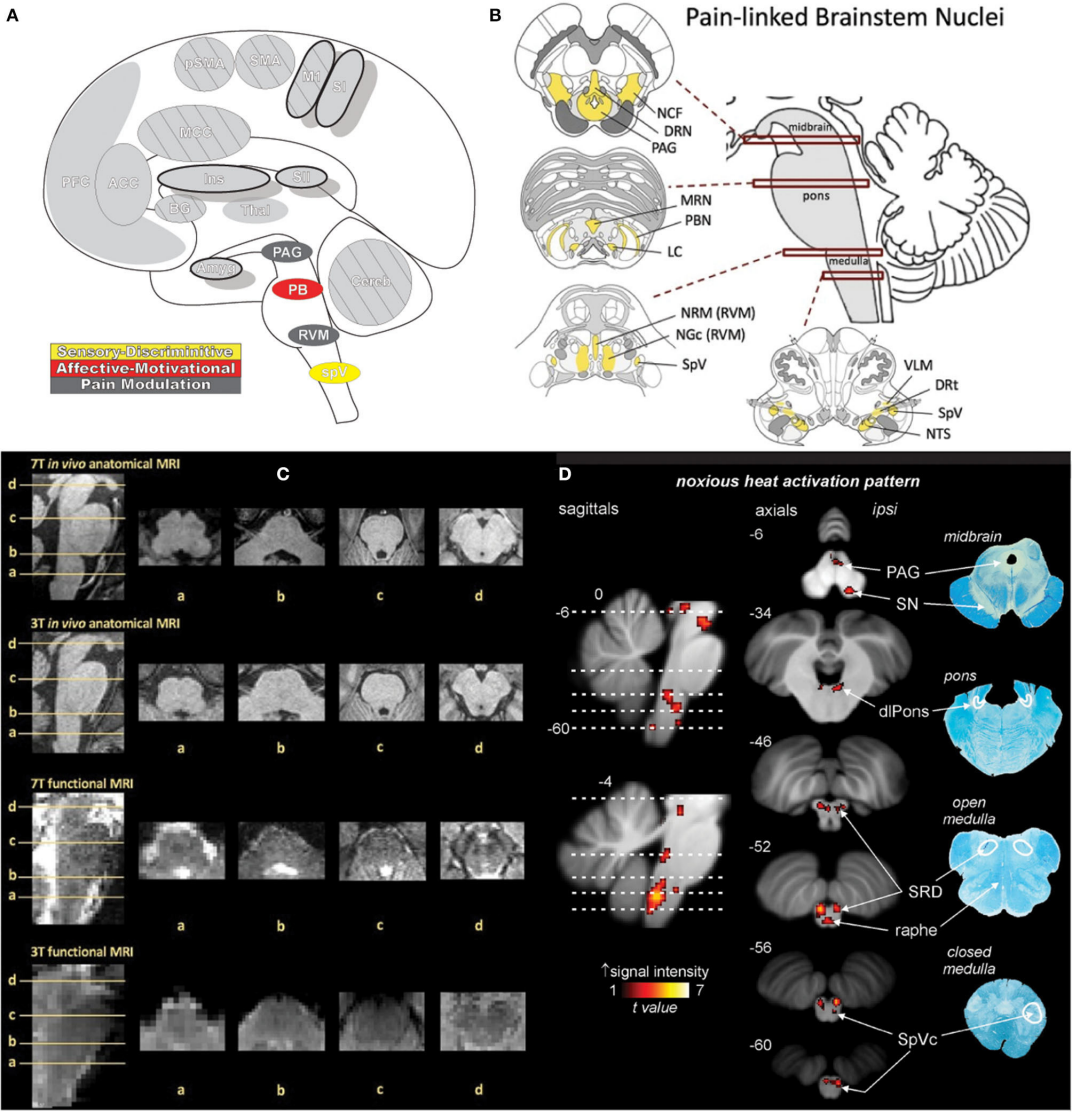
Brainstem. **(A)** Brain areas active during pain: periaqueductal gray (PAG), parabrachial nuclei (PB), rostral ventromedial medulla (RVM), spinal trigeminal nucleus (SpV) highlighted. **(B)** Schematic of brainstem nuclei associated with pain processing. Reprinted from PAIN Reports, Vol 4(4), V Napadow, R Sclocco, LA Henderson, Brainstem neuroimaging of nociception and pain circuitries, p e745, Copyright (2019) Napadow et al., under the Creative Commons Attribution License (CC-BY). DOI: https://dx.doi.org/10.1097%2FPR9.0000000000000745. **(C)** Axial slices containing brainstem nuclei from Figure. 12(B) arranged to compare the spatial resolution and quality of anatomical and functional MRI data at different magnetic field strengths (7 Tesla and 3 Tesla). Advances in imaging techniques and technologies promise to advance neuroimaging investigation of the brainstem as subtle differences in increasingly fine and detailed structures can be appreciated by MRI. Reprinted from PAIN Reports, Vol 4(4), V Napadow, R Sclocco, LA Henderson, Brainstem neuroimaging of nociception and pain circuitries, p e745, Copyright (2019) Napadow et al., under the Creative Commons Attribution License (CC-BY). DOI: https://dx.doi.org/10.1097%2FPR9.0000000000000745. **(D)** fMRI activations in the medulla, pons, and midbrain in response to brief noxious thermal stimulation, comprising activation of ascending nociceptive pathways and descending pain modulation, highlighting the dense and complex pain circuitry present in the brainstem. Myelin-stained ex-vivo axial sections are displayed to the right of corresponding sagittal and axial MRI slices. Reprinted from NeuroImage, Vol 124(Part A), AM Youssef, VG Macefield, LA Henderson, Pain inhibits pain; human brainstem mechanisms, p54-62, Copyright (2020), with permission from Elsevier. DOI: https://doi.org/10.1016/j.neuroimage.2015.08.060. DRN, dorsal raphe nucleus; DRt, dorsal reticular nucleus; LC, locus coeruleus; MRN, median raphe nucleus; NCF, nucleus cuneiformis; NGc, nucleus gigantocellularis; NRM, nucleus raphe magnus; NTS, nucleus tractus solitarii; PAG, periaqueductal gray; PBN, parabrachial nucleus; RVM, rostral ventromedial medulla; SpV, spinal trigeminal nucleus; VLM, ventrolateral medulla; SpVc, spinal trigeminal nucleus caudalis; SRD, subnucleus reticularis dorsalis; dlPons, dorsolateral pons; PAG, periaqueductal gray; SN, substantia nigra.

The brainstem (Figure 12) is a critical integrative relay between ascending inputs from primary afferents as they proceed to supraspinal areas and descending modulatory influences from supraspinal areas themselves (49). The ascending sensory system traverses the medulla, pons, and midbrain enroute to the cerebral cortex, and is modified in transit at the primary afferent synapse (i.e., spVc) as well as other brainstem regions (49, 230). Descending modulation of ascending sensory transmission is triggered by cortical and subcortical messaging to brainstem structures that can enhance or suppress the afferent signal depending on context (49, 231). That context comes in the form of situational input from amygdala, cerebellum, PFC, ACC, hypothalamus, and thalamus, which all exert “top down” pro- or antinociceptive, analgesic-mediated influence on brainstem nuclei; these circuits are referred to as the descending pain modulatory system (49, 230, 231). Bidirectional signal control is important for context-dependent pain modulation—depression of pain and antinociceptive signaling may be necessary to enact escape despite painful injury, while pronociceptive modulation promotes vigilance and protection of damaged tissue (231).

Nuclei associated with pain processing and descending modulation are situated throughout the brainstem. Within the midbrain lies the periaqueductal gray (PAG) and nucleus cuneiformis (NCF); within the pons is the parabrachial nuclei, locus coeruleus (LC), and dorsal and medial raphe nuclei; and within the medulla is the rostral ventromedial medulla (RVM), ventrolateral medulla, subnucleus reticularis dorsalis (SRD), and spV (49, 230).

PAG is a critical site in which ascending sensory and descending modulatory pathways interact and is part of the endogenous pain inhibitory system (232). In addition to pain processing and control, PAG participates in the expression of anxiety, analgesia, fear, cardiovascular function, vocalization, and reproductive behaviors (232). Diffusion tensor imaging tractography has shown PAG connections to PFC, ACC, cerebellum, hypothalamus, thalamus, nucleus accumbens, and amygdala as well as the pre- and postcentral gyri and lower brainstem nuclei, such as RVM and medullary dorsal horn (49, 232). PAG consists of four longitudinal, columnar subnuclei parallel to and surrounding the mesencephalic aqueduct: the dorsolateral (dlPAG), dorsomedial (dmPAG), lateral (lPAG), and ventrolateral (vlPAG) subdivisions (232). The subdivisions seem functionally segregated, with stimulation of lPAG and dlPAG eliciting elevated blood pressure along with active emotional coping strategies and behavioral responses (fight or flight), while vlPAG stimulation results in decreased blood pressure and passive responses (quiescence) (49, 232). Functional resting state connectivity investigations have shown additional associations and corroborated many connections identified with primate and human tract tracing, including ACC, AI, cerebellum, dorsal putamen, hippocampus, globus pallidus, and ventromedial medulla; these investigations have also revealed negative connectivity between LPFC, PI, and post-central and occipital gyri (232). In addition to functional and anatomical differences, animal studies have found fundamentally different methods of analgesia in the subregions— vlPAG is opioid-mediated while lPAG (which receives somatotopically arranged spV nociceptive projections) and dlPAG are non-opioid mediated (230, 232). Supporting these findings in animals, electrical stimulation of PAG results in analgesia that is abolished when naloxone is administered (233).

PAG has diverse brain innervation, participates in afferent sensory transmission, and, after integrating its numerous inputs, is a principal effector of the descending pain modulation system by means of its projections to the RVM and other brainstem nuclei (49, 230). RVM carries out bidirectional pro- and anti-analgesic modulation through projections to other brainstem areas, dependent on signaling from dense connections with the PAG (49, 230). RVM receives projections from parts of LC, PBN, and thalamus in addition to those from PAG, and is the lowest “common relay” of descending pain modulation pathways, subsequently sending outputs to Vc, Vi/VC, and SpV (234).

RVM can inhibit incoming noxious signals through the activation of OFF class neurons or facilitate nociception *via* ON class neurons and, at rest, the counteracting neuronal activities are thought to be balanced (230). Most information on specific cell function comes from animal studies, as the intermingled anatomy of RVM cell populations cannot be differentiated by neuroimaging (49). However, neuroimaging and resting state studies have shown increased functional coupling between vlPAG and RVM as well as RVM and multiple subnuclei of spV in patients with trigeminal neuropathy (230, 235). Animal studies of the ventrolateral medulla and NCF have reported ON/OFF cell populations, similar to those of RVM, with presumably similar functions in afferent regulation and, like the PAG, the NCF participates in ascending signal transmission and has projections to RVM (49).

Another PAG coordinated area involved in nociceptive modulation is LC, which has reciprocal connections to both vlPAG and spV in primate tracings (49, 230). LC regulates attention and mood through noradrenergic inputs to the brain while also playing a role in pain processing, such as in cognitive-mediated distraction analgesia (49). Resting state examination in painful trigeminal neuropathy has found increased resting state connectivity strength between LC and RVM, suggesting altered noradrenergic and opioid system interactions in neuropathic pain (230). Further, LC connections with the nucleus accumbens and ACC are implicated in reward signaling from pain relief, and functional connectivity between these areas is disrupted in chronic pain. The same investigation additionally found decreased connectivity between LC and vlPAG and increased LC connection strength with SRD; these results support animal models in which LC is thought to inhibit signaling in spV through direct projections and facilitate signals indirectly through connections to SRD. SRD is also involved in analgesia produced by inhibition of one stimulus by a second (conditioned pain modulation) and achieves pain inhibition by suppressing nociceptive input in spV (48, 49).

Pain-related activation in the brainstem is less commonly found in neuroimaging studies than in many brain areas, despite the documented activity in pain and nociception throughout (7, 10). The small and complex anatomy of brainstem nuclei and susceptibility to physiological noise and other distortions may mask activation along with many other technical limitations, while in other cases the intensity of experimental stimuli may not be great enough to engage the descending pain systems (49, 236).

### Thalamus

**Figure 13:**
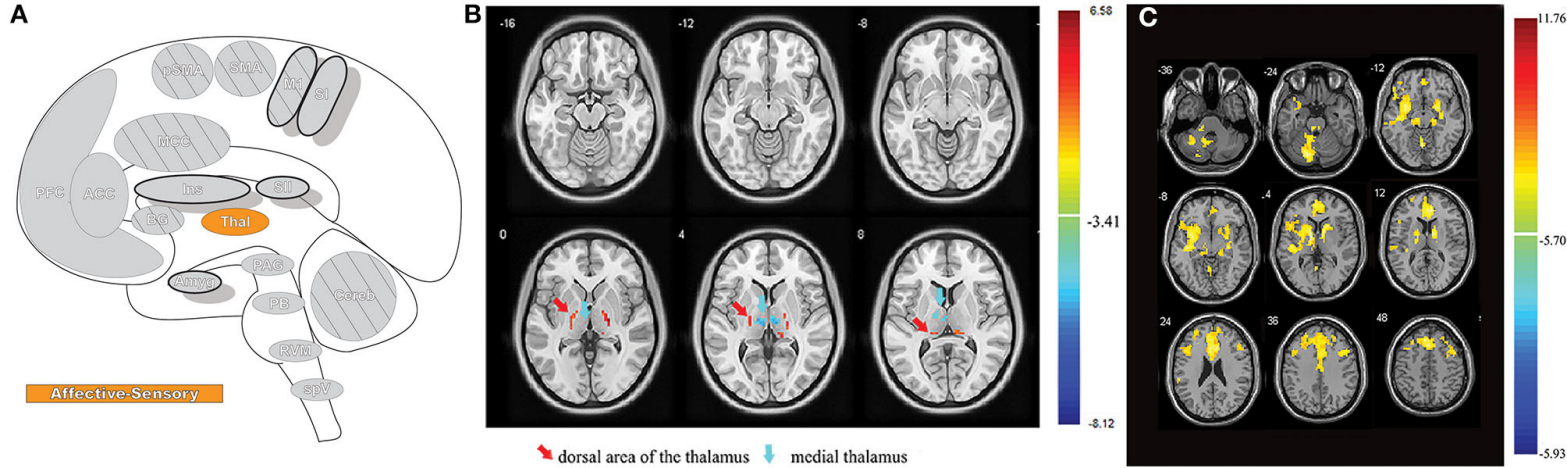
Thalamus. **(A)** Brain areas active during pain: Thalamus (Thal) highlighted. **(B)** An examination of resting state fMRI data finds that participants with orofacial pain, experimentally induced by orthodontic separators, have significantly different patterns and intensity of spontaneous neural activity (red=increased, blue=decreased) compared to controls; differences such as these support the notion that the thalamus has a greater role in somatosensory processing than simply relaying afferent signals. Reprinted from Frontiers in Neurology, Vol 11, Y Jin, H Yang, F Zhang, J Wang, H Liu, X Yang, H Long, F Li, Q Gong, W Lai, The Medial Thalamus Plays an Important Role in the Cognitive and Emotional Modulation of Orofacial Pain: A Functional Magnetic Resonance Imaging-Based Study, p589125, Copyright (2021) Jin et al., under the Creative Commons Attribution License (CC-BY). DOI: https://doi.org/10.3389/fneur.2020.589125. **(C)** The same orofacial pain participants from **(B)** were also found to have a widespread and significant reduction (yellow areas) in resting state functional connectivity between the medial thalamus and other brain areas, emphasizing the comprehensive brain-wide interconnections of the thalamus and its engagement in pain processing. Reprinted from Frontiers in Neurology, Vol 11, Y Jin, H Yang, F Zhang, J Wang, H Liu, X Yang, H Long, F Li, Q Gong, W Lai, The Medial Thalamus Plays an Important Role in the Cognitive and Emotional Modulation of Orofacial Pain: A Functional Magnetic Resonance Imaging-Based Study, p589125, Copyright (2021) Jin et al., under the Creative Commons Attribution License (CC-BY). DOI: https://doi.org/10.3389/fneur.2020.589125.

The thalamus (Thal: Figure 13) receives and passes along information from peripheral sources to the cortex, and every region of the cortex has reciprocal connections back to thalamus (237). Thalamus receives direct sensory input from numerous sources, including the trigeminothalamic tract and its connections to the ventroposteromedial (VPM) nucleus and medial nuclei, and is involved in multiple dimensions of the pain experience (238–240). Historically the thalamus was viewed as a relay site based on this widespread connectivity, but evidence continues to mount that it has a role in aggregating, processing, and integrating information from functional brain networks as well as mediating cortico-cortical connections (237, 241–243). In a view of the brain as a complex network of semi-independent modules, thalamus plays a key role in multimodal information processing by serving as an information-sharing nexus for cortical functional networks as well as structurally maintaining the modular organization of the brain network as a whole (237, 243–245).

Thalamus is composed of first-order nuclei and higher-order nuclei, which are discriminated based on the composition of their innervation—primarily ascending afferents and subcortical areas (first-order) or primarily cortical connections (higher-order) (237, 241, 242). Higher-order thalamic nuclei, through their extensive and reciprocal cortico-thalamo-cortical connections, allow for indirect interactions between areas of the cortex; first-order nuclei function more as a relay of modality-specific information to appropriate brain regions, but a role in information exchange between functionally disparate brain networks has been suggested as well (237).

Thalamus is widely activated in experimental pain and shows altered functionality in many forms of pain (7, 10, 246). Thalamic activation in response to nociceptive pain is often bilateral, another indication that the role of the thalamus goes beyond purely sensory signal transmission (7, 10). Attentional processes also increase bilateral thalamic activity, suggesting that the thalamus is involved in both discriminative and attentional networks (7).

Structural differences in thalamic gray and white matter are readily observable in numerous chronic pain studies, with evidence showing these changes may either be pre-existing or develop after exposure to pain over time (63, 247). Abnormal thalamic activation is a common finding in pain studies as well, as is aberrant spontaneous activity and accompanying burst discharge (246, 248, 249). Changes in thalamic perfusion are highly correlated with pain states, especially hypoperfusion, and deafferentation is one proposed explanation for reduced thalamic blood flow (250). However, deafferentation does not explain similar findings in patients with fibromyalgia or the fact that perfusion is often found to return to normal rates after treatment in several pain conditions (250–252). Further, a recent study of multiple sclerosis found thalamic hypoperfusion to precede atrophy of the thalamic nuclei (253).

## Pain as It Relates to Ocular Pathology and Disrupted Systems

Neuroimaging has revealed a signature of pain in the brain—a network pattern of regions activated when pain is experienced (9, 248, 254). Neuroimaging techniques have also provided a way to assess tissue thickness and gray matter density of these regions as well as their response to stimulus. Identifying the nervous system's endogenous methods of change, which may subsequently result in altered structures and functional dynamics, is key to understanding how these systems can misalign and play a role in chronic pain (63).

An important feature of the nociceptive system is its capacity for plasticity, that is, for the neurons themselves to alter their structure and function (15). Repeated or intense noxious nociception can result in sensitization “increased responsiveness of nociceptive neurons to their normal input and/or recruitment of a response to normally subthreshold inputs” (13), a synaptic plasticity that leads to signal amplification resulting in pain from normally innocuous stimuli (16). When filling an adaptive role, pain amplified in this manner helps an organism stay vigilant to the threat of further damage, and neuron thresholds normalize sometime after the initiating stimulus has been resolved (16).

### Peripheral Sensitization

Peripheral sensitization can occur when nociceptive neurons display increased responsiveness due to reduced activation thresholds and enhanced membrane excitability (13, 14, 16) and has been described previously in relation to the eye (23). The sensitized terminals of nociceptive neurons subsequently respond to stimulation that would normally be sub-noxious (allodynia) and have amplified and prolonged pain responses to noxious input (primary hyperalgesia) (14, 16, 19). Inflammatory pain is a common form of peripheral sensitization, initiated by the presence of inflammatory mediators, the release of which can be a consequence of nociceptive activity (2, 14, 44). Typically, once inflammation resolves the system returns to its previous balance, although the sensitized state can be maintained by ongoing inflammatory mediator release, thereby potentially causing neuropathic pain at the site of former injury (16, 19). Non-inflammatory causes of peripheral sensitization exist as well, as in the case of deafferentiation in postherpetic neuralgia, and the spontaneous and heightened activation of nociceptors, whatever the cause, is an important contributor to inducing sensitization in other portions of the nociceptive pathways (2, 14).

Peripheral sensitization alone cannot explain the severe level of pain in many cases. Clinical testing of abnormal pain, such as a proparacaine challenge in neuropathic corneal pain, can detect pain with a central origin; a component of pain that persists when the peripheral nociceptors have been silenced (255). Damage to underlying nerves transmitting the signal from the periphery is sometimes the cause, as when trigeminal neuropathic conditions cause pain amplification (22, 255). However, the changes observed in peripheral sensitization cannot account for several phenomena, including the temporal summation of pain, tactile allodynia, and the generation of pain by innocuous input from non-injured tissue (15, 256).

### Central Sensitization

In normal somatosensory sensation, low-intensity stimuli activate A-beta primary afferent nerves to produce non-painful sensations, despite close proximity to nociceptive pathways as the signals travel centrally to the cortex (2). The specific functional coupling of primary sensory neurons to their normal ascending pathways as well as the modularity of these parallel sensory and nociceptive circuits are determined by synaptic strength and the function of inhibitory neurons. Most input into neurons is subthreshold, but these connections are plastic, and departure from the normal balance of excitation and inhibition can cause exaggerated and abnormal pain.

Central sensitization is classified as “increased responsiveness of nociceptive neurons in the central nervous system to their normal or subthreshold afferent input. This may include increased responsiveness due to dysfunction of endogenous pain control systems” (13). Molecular, cellular, and anatomical changes can contribute to functional alterations in ocular central pain pathways, from the level of trigeminal brainstem to thalamus and up through cortical endpoints (13, 16, 22, 44, 84). Altered responsiveness in central sensitization is caused by enlarged receptive field sizes, increased membrane excitability, alteration of temporal firing dynamics, facilitated synaptic efficacy, or reduced inhibition in the neuronal circuitry of the ocular somatosensory system (16, 44). Sensitization of central neurons is often use-dependent, in which repeated activation triggers the change in synaptic functioning, and can be divided into either homosynaptic or heterosynaptic potentiation (15, 16).

Homosynaptic potentiation occurs when repeated use of a synapse facilitates subsequent activation in the same pathway, amplifying future instances of the same input (16, 257). This process is not unique to central sensitization in pain. Long Term Potentiation (LTP), the presumed hippocampal mechanism of memory, is a result of persistent homosynaptic facilitation (16). Windup is a transient form of homosynaptic potentiation in which the delivery of identical, repetitive, low-frequency noxious input results in each additional stimuli generating a larger action potential (and greater pain), but synaptic excitation returns to baseline within seconds after stimulation has ceased (16). Central sensitization remains autonomous for hours after induction and manifests after the triggering stimulus, unlike windup, which occurs during stimulation (256). Abnormal activity that triggers central sensitization by repeated nociceptive pathway use (i.e., peripheral sensitization, windup, or ectopic bursts) can cause an LTP-like homosynaptic effect (16). Future engagement of the system is facilitated, and the strengthening of synapses leads to an increase in the frequency and size of postsynaptic action potentials in TBNC (16, 44, 84). Homosynaptic functional alterations in central sensitization are contributors to primary hyperalgesia, along with peripherally sensitized areas (16).

Heterosynaptic potentiation occurs when activity in a synapse of a pathway enhances signaling in nearby, uninvolved synapses in the neuron (16). Enhancement of nearby synapses underpins the generation of pain by non-nociceptive stimuli; these heterosynaptic changes are the cause of sensitivity and pain spreading to uninjured areas (secondary hyperalgesia) as well as pain resulting from the activation of low-threshold input (allodynia) (16). In addition to the enhancement of transmission between trigeminal neuron axons and the TBNC, evidence of synaptic plasticity has been found in ACC, amygdala, PFC, and the PAG (19).

Central sensitization is a long-lasting endogenous process triggered by nociceptor input that eventually resolves when no abnormal signaling is present but can be maintained by low levels of stimulation (2). Ongoing ectopic pain (as can manifest after LASIK) or persistent peripheral sensitization (such as inflammatory dry eye disease) thus can have a role in both the generation and maintenance of central sensitization, altering the central nervous system response to stimulus for as long as the nociceptive signals persist (12, 44, 84, 257). The eventual de-escalation of the heightened pain response and reset of synaptic excitability is thought to be accomplished by inherent compensatory responses in descending pain modulatory pathways (44, 258). Damage or dysfunction in ascending pathways and descending pain modulatory processes can lead to pathological chronification of the hyper-responsive state, regardless of peripheral input. Long term maintenance of a sensitized state can be found underlying several painful craniofacial conditions and an out-of-balance descending system, especially one that promotes descending pain facilitation, can contribute to or sustain long term centralized neuropathic pain (6, 13, 14, 16, 234, 259).

### Reorganization of Functional Networks

Supraspinal areas associated with pain are functionally intertwined in their activity, allowing changes in one structure to affect larger groupings of brain areas, casting doubt on some previous models of the pain-stimulus relationship (260–262). Identifying differences in structure and function in one supraspinal area may explain larger-scale patterns of change across the brain, highlighting the inherent connectivity between brain regions that work in concert.

Synchronized rhythmic fluctuations of activity in the brain measured as fMRI signal oscillations indicate the transfer of information between regions and give insight into how supraspinal areas are joined together as part of a network (245, 263, 264). While beyond the scope of this review, these network interactions are one of the largest areas of focus in modern pain research (262, 265). Network relationships can be quantified, and differences can be seen in many disease and pain states (9, 266). These altered structural, functional, and network-associative changes in neuronal processing centers can result in amplified pain beyond the afferent signal transmitted by the periphery, leading to hyperalgesia, allodynia, and even spontaneous pain (63, 262, 266–269).

### Neuroimaging Supraspinal Eye Pain- Qualifications and Clinical Adaptation

Neuroimaging in clinical ophthalmology is often limited to interrogating CNS causes of vision loss, nystagmus, ptosis, proptosis, diplopia, ophthalmoplegia, or optic nerve abnormalities. These may or may not be accompanied by pain (270). When pain is present it can be debilitating as the eye, and craniofacial region as whole, is subject to some of the most frequently diagnosed and intense pain conditions (234). As the eye is a critical sensory structure, the associated pain can come with intense psychological, emotional/affective components (234). The most common cause of neurological eye pain is migraine, followed by primary headaches, and trigeminal pain conditions, however, most neurological disorders can lead to referred eye pain (271). In the majority of these cases neuroimaging is not recommended unless a lesion or other underlying pathology mimicking these conditions is suspected (271). Similarly neuroimaging in patients with normal ophthalmic examinations as a pain diagnostic often does not provide a clear answer to symptoms, and applying experimental results to clinical pain realities often finds a much less direct relationship between pain and stimulus (271, 272).

The variability of pain activations between individuals can be extreme, even in an individual it is difficult to predict pain, as numerous brain regions modify sensory input along with psychological and attentional processes (6, 272). Further, no brain networks or regions associated with pain are exclusively pain related and most painful situations also engage other networks and processes, like attention, emotion and salience; the resulting overlap in brain regions active in pain and other salient experiences makes pain-specific imaging biomarkers difficult to determine (261, 272–274). Even chronic pain-associated processes and abnormalities are not pain-specific and have been observed in other conditions such as anxiety and depression (272, 273). Thus, despite evidence that certain regions are reliably activated in response to noxious stimulation, the adoption of brain imaging as a direct facsimile for pain, an inherently subjective experience, is not established (272, 273, 275).

**Figure 14:**
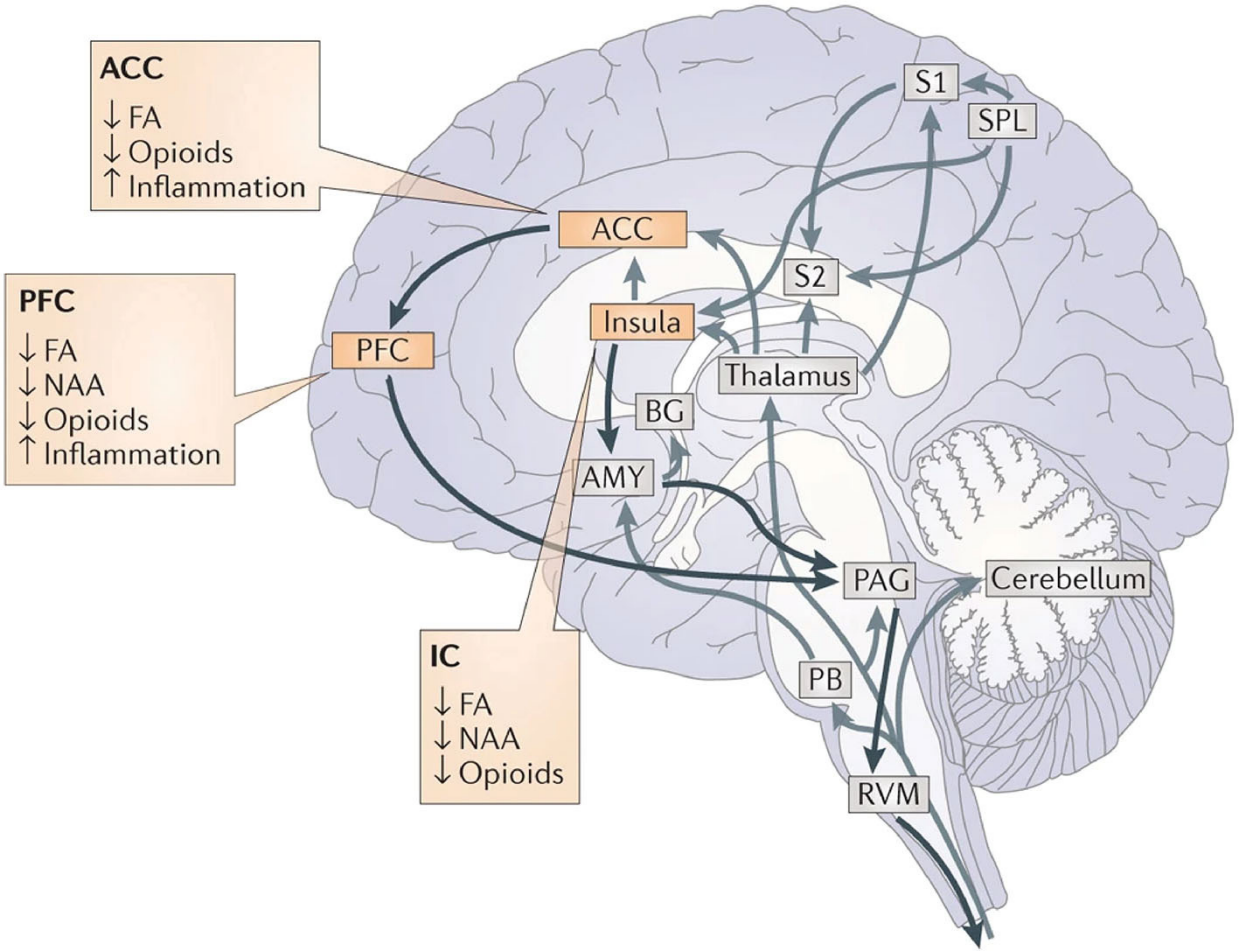
Consistent patterns of chronic pain. The ACC, PFC, and IC consistently display decreased grey matter in chronic pain conditions, along with impaired white matter health (FA) and opioid receptor binding. Chronic pain is also associated with reductions in N-acetyl aspartate in the ACC and PFC, while studies in rodents have found increased inflammation in these regions as well. Reprinted with permission from Springer Nature Customer Service Centre GmbH: Springer Nature, Nature Reviews Neuroscience, Vol 14, Cognitive and emotional control of pain and its disruption in chronic pain, MC Bushnell, M Ceko, LA Low, p502–11, Copyright (2013). DOI: https://doi.org/10.1038/nrn3516. Grey arrows, pain pathways; Black arrows, descending pathways; FA,functional anisotropy: a measure of molecule diffusion that serves as an index of white matter integrity; Opioids, opioid receptor binding: a marker of the ability to bind opioids and a way to analyze the health of descending pain systems; NAA, N-acetyl aspartate: a marker of neuronal viability. ACC, anterior cingulate cortex; PFC, prefrontal cortex; IC, insular cortex; S1, primary somatosensory cortex; S2, secondary somatosensory cortex; SPL, superior parietal lobe; BG, basal ganglia; AMY, Amygdala; PAG, periaqueductal gray; PB, parabrachial nuclei; RVM, rostral ventromedial medulla.

In addition to the overlap in regional and network functions, multiple factors can influence painful stimuli-induced activations that may affect the ability to directly translate experimental work into the clinic and thus hinder the adoption of neuroimaging as a formal standard of care. Biological sex has a significant effect on pain-related brain activations, as studies have reported variance in experienced and anticipatory pain between women and men, along with differential activations in the ACC, insula, and parietal and somatosensory cortices, among other areas (275, 276). Other factors contributing to variation in the neuroimaging of pain, although not an exhaustive list, include the duration of the painful stimulus, type of pain, mechanisms of pain, and type of disease underlying the pain; different patterns of activation can be observed in allodynia and hyperalgesia compared to normal individuals, mechanical and thermal pain have stronger or weaker activations in some brain areas when compared, and different diseases may have no functional similarities aside from increased pain thus making the results extremely specific to each condition and not widely appliable (272, 275). Despite these issues, some studies have found patterns of differential activations and even structure in some brain areas that are consistent across conditions (6) (Figure 14), however, other analyses find no consistent abnormalities in fMRI responses to painful stimuli in chronic pain patients- likely due to a combination of factors described above and many other uncorrected differences in experimental criteria that must be accounted for to achieve meaningful clinical translation (20, 277). While concrete and universal pain imaging biomarkers are yet to be established, neuroimaging has identified key brain regions involved in acute pain and established that CNS function is disturbed in chronic pain (272, 274).

## Conclusion

The principles for supraspinal encoding of eye pain are akin to those observed in the rest of the body. Pain is a subjective experience that engages a concert of multidimensional processes throughout the brain. Different areas encode distinct aspects of sensory and emotional processes as well as the cascade of reactive autonomic, cognitive, reflexive, and modulatory mechanisms relevant to protective behaviors and adaptation. In relation to eye pain, the brain receives afferent input from the trigeminal system, which it also modulates using descending cortico-medullary feedback and feed-forward loops. Further active areas of investigation include the transformation of acute pain to chronic pain, improved characterization and differentiation of brain networks in chronic pain conditions, sex differences in the processing of pain, interactions between the immune system and brain regions, and patient stratification for targeted therapies for specific chronic pain conditions (278).

## Author Contributions

EM conceived of and directed the manuscript. NP drafted the initial manuscript. NP and EM wrote the final manuscript. All authors contributed to the article and approved the submitted version.

## Conflict of Interest

The authors declare that the research was conducted in the absence of any commercial or financial relationships that could be construed as a potential conflict of interest.

## Publisher's Note

All claims expressed in this article are solely those of the authors and do not necessarily represent those of their affiliated organizations, or those of the publisher, the editors and the reviewers. Any product that may be evaluated in this article, or claim that may be made by its manufacturer, is not guaranteed or endorsed by the publisher.
